# Ecologically informed microbial biomarkers and accurate classification of mixed and unmixed samples in an extensive cross-study of human body sites

**DOI:** 10.1186/s40168-018-0565-6

**Published:** 2018-10-24

**Authors:** Janko Tackmann, Natasha Arora, Thomas Sebastian Benedikt Schmidt, João Frederico Matias Rodrigues, Christian von Mering

**Affiliations:** 1 Institute of Molecular Life Sciences and Swiss Institute of Bioinformatics, University of Zurich, Zurich, Switzerland; 2 Zurich Institute of Forensic Medicine, University of Zurich, Zurich, Switzerland; 3 Present address: European Molecular Biology Laboratory, Heidelberg, Germany

**Keywords:** Human microbiome, Biomarkers, Mixture, Random Forest, Generalized Local Learning

## Abstract

**Background:**

The identification of body site-specific microbial biomarkers and their use for classification tasks have promising applications in medicine, microbial ecology, and forensics. Previous studies have characterized site-specific microbiota and shown that sample origin can be accurately predicted by microbial content. However, these studies were usually restricted to single datasets with consistent experimental methods and conditions, as well as comparatively small sample numbers. The effects of study-specific biases and statistical power on classification performance and biomarker identification thus remain poorly understood. Furthermore, reliable detection in mixtures of different body sites or with noise from environmental contamination has rarely been investigated thus far. Finally, the impact of ecological associations between microbes on biomarker discovery was usually not considered in previous work.

**Results:**

Here we present the analysis of one of the largest cross-study sequencing datasets of microbial communities from human body sites (15,082 samples from 57 publicly available studies). We show that training a Random Forest Classifier on this aggregated dataset increases prediction performance for body sites by 35% compared to a single-study classifier. Using simulated datasets, we further demonstrate that the source of different microbial contributions in mixtures of different body sites or with soil can be detected starting at 1% of the total microbial community. We apply a biomarker selection method that excludes indirect environmental associations driven by microbe-microbe associations, yielding a parsimonious set of highly predictive taxa including novel biomarkers and excluding many previously reported taxa. We find a considerable fraction of unclassified biomarkers (“microbial dark matter”) and observe that negatively associated taxa have a surprisingly high impact on classification performance. We further detect a significant enrichment of rod-shaped, motile, and sporulating taxa for feces biomarkers, consistent with a highly competitive environment.

**Conclusions:**

Our machine learning model shows strong body site classification performance, both in single-source samples and mixtures, making it promising for tasks requiring high accuracy, such as forensic applications. We report a core set of ecologically informed biomarkers, inferred across a wide range of experimental protocols and conditions, providing the most concise, general, and least biased overview of body site-associated microbes to date.

**Electronic supplementary material:**

The online version of this article (10.1186/s40168-018-0565-6) contains supplementary material, which is available to authorized users.

## Background

The identification of microbial biomarkers and their use for classification provide valuable information for predictions in a wide range of applications. For example, disease states can be associated with complex microbial patterns, making suitable biomarker detection and classification methods crucial for accurate disease identification and prediction [1–4]. Another important application is the identification of the source of origin for a sample, for example in forensic cases [5] or environmental monitoring [6]. In the former, reliably determining the bodily source of a stain at a crime scene (e.g., saliva, semen, vaginal fluid, blood) can critically aid the reconstruction of crime events. In the latter, distinguishing microbial communities of human origin from those of environmental provenance can be of great value, for example in studies of human sewage pollution [7]. Establishing the source of microbial communities has an added complexity when dealing with mixtures, such as in contaminated samples, as these require the distinction of two or more different sources.

In the last decade, advances in high-throughput sequencing have led to a marked increase in both the number and sequencing depth of human microbiome studies, encompassing a wide range of experimental protocols and conditions (e.g., sex, geography, medication) [8, 9]. This increase in data volume and diversity has opened the door for human microbiome studies to apply more advanced statistical and machine learning tools [10, 11].

Pioneering studies have explored the potential of supervised machine learning approaches on a number of microbiome-based classification tasks, such as identification of individual subjects, disease prediction, and body site identification [10–12], mostly focusing on comparatively small-scale datasets from individual studies [13, 14]. In these evaluations, the best-performing methods generally achieved high predictive power, with Random Forest Classifiers (RFCs, [15]) ranking consistently among the top performing models. Further studies have successfully applied classification methods to varying sample types such as human, soil, and sediments [16–18] and also in the context of multi-source mixtures [19, 20].

While the success of supervised learning methods has been demonstrated in individual studies, it is largely unclear whether these results generalize to a meta-study setting. For instance, a large fraction of publicly available data consists of amplicon sequencing runs, where primer specificity can result in amplification biases [21, 22]. This can affect taxonomic inferences and classification accuracy, potentially leading to classifiers with top performance for some primer types and mediocre performance for others. While metagenomic shotgun sequencing tends to produce less biased estimates of community composition, this approach is more expensive and typically results in a lower coverage of the 16S rRNA gene, potentially compromising the resolution of abundances for rare taxa [23]. Apart from protocol-specific effects, subject-specific factors such as geographic location and medication may introduce further biases. Recent work in disease prediction also suggests that individual studies may report unspecific signals only properly appreciated when aggregating studies [24].

The aggregation of sequencing data from different studies into large meta-datasets and their utilization for classifier training could thus lead to more general and predictive models that can reliably classify samples produced under a variety of experimental protocols and from a wide range of subjects. It further may allow identification of biomarkers that overcome biases of individual studies. Recent work by Pasolli et al. [25] showed promising results for the predictive power of cross-study models for classification of diseased versus healthy subjects, as well as classification of body sites. However, their body site classification analysis was based on 642 sequencing samples from only two studies and furthermore restricted to whole genome shotgun sequencing. Therefore, it is yet unclear whether these results generalize across larger and experimentally more heterogeneous datasets.

Such datasets are of particular relevance for the identification of microbes endemic to human body sites. Site-specific microbes have been extensively studied in large cooperative efforts, such as the Human Microbiome Project (HMP, [26]), and in smaller studies by individual groups. However, single-study biases and insufficient sample size could significantly influence previously reported associations. For instance, the original HMP was restricted to 242 healthy adults situated in the USA and limited to two primer sets, raising questions about whether reported site-specific taxa generalize across geography, subject-specific conditions, and experimental protocols.

While some microbes are endemic to a body site, others are only indirectly associated with a site due to their ecological dependency on endemic microbes. These indirectly associated microbes could in principle also thrive in other habitats, where the same requirements may be fulfilled by other partners. Common biomarker identification approaches may misinterpret this ecological signal and specify such indirectly associated microbes as body site biomarkers. More refined methods, on the other hand, can separate directly and indirectly associated markers by testing whether an association signal can be explained by other variables [27, 28]. Generalized Local Learning (GLL, [27]), a method that excludes indirectly associated biomarkers, was previously shown to achieve the best balance between number of identified biomarkers and accuracy in psoriasis prediction [12]. Importantly, such ecological interaction effects were usually not considered by previous studies on biomarkers for human body sites, potentially inflating numbers of reported markers.

In this study, we had two aims: (i) to train a supervised classification model on a heterogeneous, large-scale dataset and evaluate its performance compared to a single-study classifier in body site prediction, both for single-source samples and mixtures, and (ii) to obtain a high-quality set of microbial biomarkers directly associated with human body sites, excluding microbe-microbe-driven associations. To this end, we retrieved 50,273 publicly available sequenced samples from five human body sites (skin, saliva, vagina, nostril, and feces), further filtered down to 15,082 classification-ready samples spanning 57 studies. We additionally retrieved sequencing data for 1329 soil samples as representatives of a typical environmental contaminant. We used the body site dataset for classifier training and evaluated its performance on single-source samples, as well as in silico mixtures of samples from two body sites or from a body site and soil. We compared this performance to a classifier trained on a single study, subject to previous machine learning benchmarks, and demonstrated that the cross-study classifier makes strongly improved predictions. Finally, we identified a parsimonious core set of microbial biomarkers for the investigated body sites, which included previously unreported biomarkers and, mostly due to our bias-mitigating cross-study approach and the exclusion of indirect associations, rejected previously reported study-specific associations. We analyzed this set of biomarkers in depth in terms of taxonomy, phylogeny, and physiological traits.

## Results

### A large and heterogeneous collection of microbial sequencing samples from human body sites

**Fig. 1:**
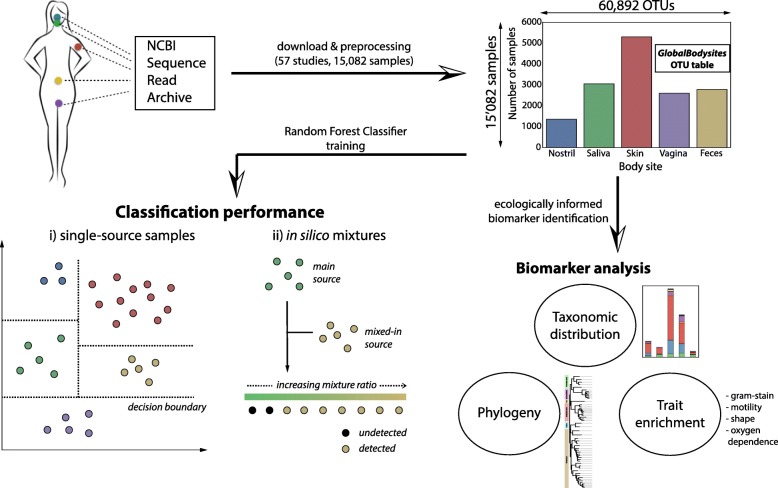
Fifteen thousand eighty-two samples from five body sites and 57 studies were queried from the NCBI Sequence Read Archive and uniformly processed, yielding the GlobalBodysites dataset. A Random Forest Classifier (RFC-global) was trained and evaluated on this dataset, followed by identification and analysis of ecologically informed microbial biomarkers

Publicly available metagenomic sequence read data were retrieved from the NCBI Sequence Read Archive [29] for studies investigating microbial communities in five human body sites: nostril, saliva, skin, vagina, and feces. The initial dataset consisted of 50,273 samples, sequenced mainly through targeted amplicon sequencing and whole genome shotgun sequencing technology. After extensive filtering (see the “Methods” section), this dataset was reduced to a condensed set of 15,082 samples (see Additional file 1: Table S4 for accession numbers) from 57 studies, where the number of samples per body site ranged between 1354 (nostril) and 5296 (skin). In total, 60,892 operational taxonomic units (OTUs) were identified after mapping to a database of 16S rRNA reference sequences provided by MAPseq [30], pre-clustered at 96% sequence similarity (Fig. 1). We refer to this dataset as GlobalBodysites.

### Cross-study classifier outperforms single-study model in predictive accuracy

**Fig. 2:**
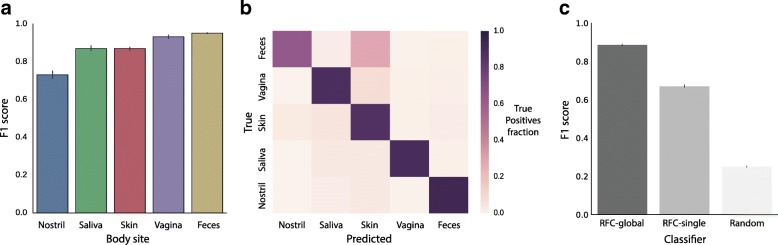
**a** Prediction performance of RFC-global was estimated on five cross-validation sets of unseen samples, measured as F1 score. **b** Distribution of true positive and false positive classifications across all pairs of body sites. **c** Comparison of RFC-global to a RFC trained on only samples from the Costello et al. dataset (RFC-single) and a random guesser, measured as F1 score

We trained an optimized Random Forest Classifier (RFC-global) on the GlobalBodysites dataset and assessed its performance on labelling samples with their correct body sites in a fivefold cross-validation framework (see the “Methods” section). Performance was measured through F1 scores, which take into account both precision and recall and are less affected by imbalanced numbers of samples per body site than other metrics. The classifier was able to accurately identify body site labels in the cross-validated test sets (Fig. 2a), with mean F1 scores between 0.73 (nostril) and 0.95 (feces). Training and testing the classifier on sample sets with biased body site proportions yielded comparable results (Additional file 2: Figure S10b).

We next investigated which pairs of body sites were most challenging to distinguish for RFC-global. To this end, we generated a confusion matrix, capturing the misclassifications across all pairs of body sites (Fig. 2b). We observed a relatively small number (5 to 11%) of misclassifications for all site pairs except one: nostril samples were more prone to misclassification (36%), with a majority of mislabellings as skin (33%). This pattern is in line with nostril-skin misclassifications observed in previous work [25].

Next, we compared the predictive performance of RFC-global to a classifier trained only on the samples from a single study. To this end, we trained a RFC on the subset of GlobalBodysites comprising 372 samples from Costello et al. [13] (RFC-single), a dataset used extensively for body site prediction benchmarks in previous research [10, 11]. We found the prediction performance for RFC-global (trained across studies) to be considerably higher than that for RFC-single (mean F1 of 0.89 compared to 0.66, an increase of 35%) (Fig. 2c).

In order to further elucidate this difference in predictive performance, we looked at the intrinsic feature importance assignments of the RFCs in detail. Briefly, RFCs assign a weight (feature importance) to each OTU depending on its estimated predictive importance. We found that 91.4% of the 1397 predictive OTUs (feature importance > 0) reported by RFC-single were also supported by RFC-global. However, RFC-global reported 15,863 additional predictive OTUs (Additional file 3: Figure S1a,b), an increase by a factor of more than 12. Additionally, 8.6% of the OTUs predictive for RFC-single were dismissed as uninformative by RFC-global. We further observed that feature importances of overlapping predictive OTUs were only moderately correlated (Spearman’s rho of 0.68, *p* value < 0.05, Additional file 3: Figure S1c).

In order to estimate whether increased prediction performance extends to other single studies, we further trained a classifier on all 5433 samples belonging to the Human Microbiome Project (HMP, RFC-single-hmp), which constitutes the largest single study in GlobalBodysites (36% of all samples). While performance of RFC-global was closer to RFC-single-hmp (12% F1 score increase, Additional file 2: Figure S10a, “unweighted”), we found this greater similarity to be driven by the dominant fraction of HMP samples in our validation sets. When weighting studies by the inverse of their sample numbers in F1 score calculation, effectively increasing the reward for correctly predicting samples from smaller studies, we found the performance increase of RFC-global to be noticeably more pronounced (41% F1 score increase, Additional file 2: Figure S10a, “weighted”).

We next investigated whether the performance of RFC-global could benefit from additional data. To this end, we tested classification performance for increasing numbers of studies and samples, starting with only the HMP project (Additional file 4: Figure S11). For the “unweighted” scenario (see previous paragraph), we found that performance plateaued around 30 studies (~ 8000 samples), indicating small benefits from additional studies for classifier performance. However, when increasing the reward of predicting samples from smaller studies (“weighted”), we found that performance did not reach a plateau but instead consistently increased with more studies.

### Even trace amounts of body site microbiomes can be reliably identified in mixtures between body sites or body site and environment

Next, we evaluated detection limits and prediction performance of RFC-global on in silico mixtures of different body sites (see the “Methods” section). The task was to identify a microbial community from a target body site in mixtures of communities from the target body site and a background body site, along a gradient of increasing mixture fractions, for all pairs of body sites. Classification performance was measured through the area under the ROC curve (AUC) metric, which similarly to F1 scores is robust to label imbalances, but quantifies predictive performance independent of a decision threshold.

**Fig. 3:**
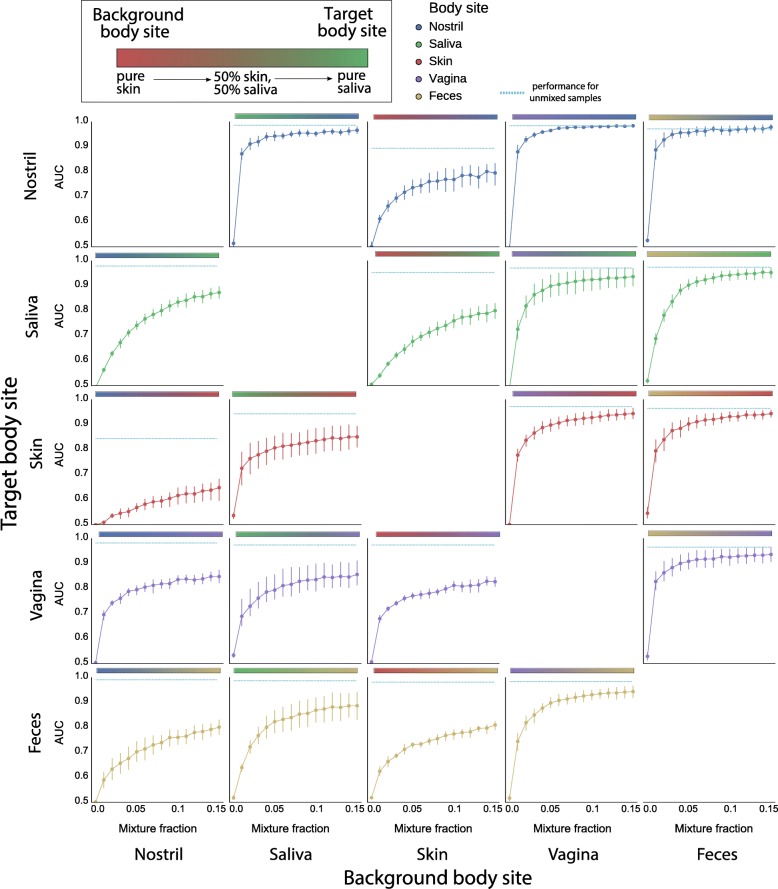
Discrimination performance of RFC-global on mixed samples with varying levels of mixture fractions (up to 15%). Mixtures of all pairs of body sites were prepared using an in silico procedure (see the “Methods” section) and then predicted by RFC-global. Prediction performance was quantified in terms of AUC. Dotted lines indicate performance evaluated on unmixed samples

At 15% mixture fraction, RFC-global achieved more than 75% of the AUC obtained for unmixed samples in 15 out of 20 body site combinations (Fig. 3). For six of these combinations, these AUC scores were achieved even at trace amounts as low as 1 to 2% mixture fraction. Highest performance was reached when the source body site was vagina or feces, with an average AUC of 0.81 at 2% mixture fraction. In agreement with the confusion matrix of single-source samples (Fig. 2b), distinguishing skin and nostril samples was challenging: mixtures containing these two body sites required at least 70% skin for the AUC to reach 0.8 when skin was the target body site (Additional file 5: Figure S2). Interestingly, the identification of nostril in mixtures containing skin required a mixture fraction of only 30% to achieve the same AUC.

Compared to RFC-global, RFC-single was considerably less robust to mixed sources: predictions resulted in lower AUC values irrespective of which body sites were mixed, in some cases down to little more than random guessing (AUC < 0.6), even when the target body site comprised up to 80% of the mixture (Additional file 6: Figure S4).

Since AUC measures general discriminative power without setting a specific classification threshold, we also determined thresholds above which the presence of a body site fraction in a mixture was likely. To this end, we estimated optimal prediction thresholds for all body site combinations based on the training data and computed F1 scores for each pair of body sites (Additional file 7: Figure S3). For most pairs, F1 results were comparable to the AUC analyses, but some combinations required higher fractions of the target body site when imposing a fixed threshold.

We next investigated the predictive performance and robustness of our classifier in mixtures comprising bacterial communities from a human body site and an environmental component. We prepared in silico mixtures between body site samples from the GlobalBodysites data and 1329 additional microbial soil samples from the NCBI SRA database. For all non-fecal mixtures, we obtained AUC values greater than 0.9 even in samples that consisted mostly of soil (body site mixture fractions below 10%) (Additional file 8: Figure S5). For feces, performance was slightly decreased to between 0.8 and 0.9 AUC for mixture fractions below 50%.

In order to also test the robustness of the classifier to contamination in training samples, we randomly mixed 50% of all training samples with 30% soil, followed by classifier training (RFC-global-contaminated). Validation of RFC-global-contaminated on unmixed body site samples resulted in F1 scores similar to RFC-global (mean decrease 1.3%).

### A parsimonious core set of directly associated microbial biomarkers for human body sites

Having assessed the predictive power of RFC-global, we were next interested in biological patterns driving its performance and whether new or unusual associations between microbes and environment could be detected in GlobalBodysites. We thus sought to identify a core set of microbial biomarkers for each investigated body site.

**Fig. 4:**
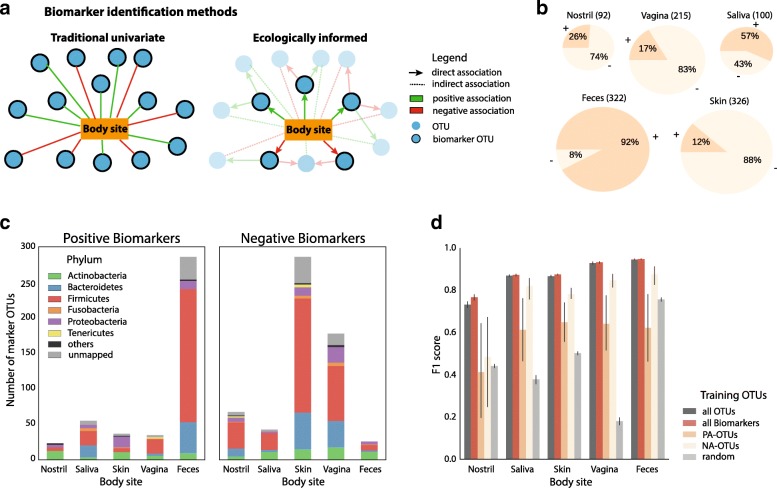
Identified ecologically informed biomarker OTUs. **a** Comparison of traditional univariate and ecologically informed biomarker identification. Ecological association information can be used to discard biomarkers indirectly associated with body sites. **b** Between 92 and 326 positively and negatively associated biomarkers were identified per body site. **c** Phylum-level distribution of positive and negative biomarkers per body site. **d** Predictive performance of classifiers for each body site, trained on different sets of OTUs: all OTUs, all biomarkers, only PA-OTUs (positive biomarkers), only NA-OTUs (negative biomarkers), and a random set of OTUs of size equal to the all biomarkers

We used Generalized Local Learning (GLL, [27]) (Fig. 4a), an approach that has advantages over feature importances reported by Random Forests and decision trees ([31], see Methods).

Briefly, GLL only reports OTUs as biomarkers whose association with a habitat cannot be statistically explained by ecological dependencies on other OTUs in that habitat. This effectively makes the identified biomarker sets more parsimonious, as OTUs found to be only indirectly associated with a habitat are excluded.

In our study, GLL reduced the number of OTUs from an initial 60,982 to 635 directly associated biomarkers, between 92 (nostril) and 326 (skin) (Fig. 4b, Additional file 9: Table S5). When evaluating single-source samples, the predictive performance (F1 score) of a RFC trained only on biomarkers (RFC-global-GLL) was slightly increased (1 to 5%) compared to RFC-global (Fig. 4d). In contrast, a random subset of all OTUs with size equal to the biomarker set resulted in strongly reduced F1 scores that ranged from a 20% reduction in feces up to an 80% reduction in vaginal sites (Fig. 4d).

We further evaluated the performance differences between RFC-global versus RFC-global-GLL in simulated mixtures of body site and soil samples and found that biomarker-only RFC models performed worse on such mixtures, with decreases that ranged between 50% AUC for feces and 5% for vaginal sites (Additional file 8: Figure S5).

In order to test how GLL biomarker discovery was affected by dataset size, we further applied it to the Costello et al. dataset. This test yielded 12 biomarkers (98% less than GlobalBodysites) which achieved an average F1 score of 0.39 (compared to 0.89 in RFC-global-GLL, Additional file 10: Figure S6a).

### Negatively associated microbes are numerous and contribute strongly to sample prediction accuracy

Among the directly associated biomarkers selected by GLL, we found both positively associated and negatively associated OTUs (PA-OTUs and NA-OTUs, Fig. 4a, b). The presence of PA-OTUs associated with a body site in a sample increased the likelihood of classification as that body site, while their absence decreased it (vice versa for NA-OTUs). We observed a large fraction of PA-OTUs for feces (92%), while for nostril, skin, and vagina samples, mostly NA-OTUs were identified (74 to 88%). For saliva, fractions of positively and negatively associated OTUs were balanced (57% PA-OTUs). Average prevalence—the number of samples a biomarker OTU is found in across all body sites—was slightly elevated for NA-OTUs (Additional file 11: Figure S9).

All pairs of body sites showed a varying amount of overlap among identified biomarker OTUs (Additional file 12: Figure S7). Notably, this overlap was in most cases oppositional (positive for one body site, negative for the other). An exception to this pattern was the combination nostril-skin, for which 98% of shared biomarker OTUs had the same association type.

We compared the importance of the PA-OTUs and NA-OTUs in classification accuracy by training RFC classifiers separately on only positively or negatively associated biomarkers. When using PA-OTUs only, we observed 25–55% lower F1 scores (for skin and nostril, respectively). In contrast, using only NA-OTUs resulted in smaller decreases in F1 score ranging between 4% for saliva to 11% for skin for all sites except nostril which showed a reduction of 35% (Fig. 4d).

### Previously unreported associations between microbes and body sites

We next examined the taxonomic profiles of detected biomarker OTUs. These OTUs stemmed almost exclusively from the domain *Bacteria*, with a small number of *Archaea* and *Eukaryota* (0.3% and 0.5% respectively). As shown in Fig. 4c, four phyla were dominant in the selected biomarkers across all body sites: *Bacteroidetes*, *Firmicutes*, *Proteobacteria*, and *Actinobacteria*. With few exceptions, both PA-OTUs and NA-OTUs from these phyla were found across all body sites. We found high fractions of positive *Firmicutes* and *Bacteroidetes* biomarkers in feces and *Proteobacteria* biomarkers in skin. Furthermore, *Tenericutes* included predominantly PA-OTUs for vagina and NA-OTUs for skin and nostril.

We identified 107 distinct genera among all 635 biomarker OTUs. Numerous associations found in our automated analysis were in line with previously reported associations (e.g., *Corynebacterium* and *Cutibacterium* for skin, *Lactobacillus* for vagina, *Bacteroides* for feces, and *Prevotella* for saliva; see Additional file 13: Table S1 and Additional file 14: Table S2). Nonetheless, we also detected novel specific genus-body site associations that, to our knowledge, have not been discussed elsewhere (Table 1): *Ralstonia* and *Caulobacter* were found to be directly associated with skin, *Delftia* to nostril, and an archaeal OTU mapping to the genus *Halovenus* to feces. It is furthermore noteworthy that GLL marked a number of previously reported genera as only indirectly associated in our study, for instance *Megasphaera* in saliva [32], *Veillonella* in skin [26], *Mobiluncus* in vagina [33], and *Bacillus* in feces [34].

**Table 1:** Novel positively associated biomarker genera for each body site. Weight of each biomarker is measured by the percentile of feature importance in RFC-global amongst biomarker OTUs of the same body site. Prevalence is the average number of samples that biomarker OTUs of a genus were found in across all body sites

Body site	Genus	Prevalence	Percentile of Random Forest feature importance
Nostril	*Delftia*	2480	83.0
Skin	*Ralstonia*	2666	83.7
	*Caulobacter*	1280	71.2
Feces	Putative *Halovenus*	266	0.1

Across all genera that comprised the 635 identified biomarker OTUs, 14 included PA-OTUs for multiple body sites. For instance, *Actinomyces* contained PA-OTUs for saliva, skin, and vagina. Moreover, five genera included both PA-OTUs and NA-OTUs for the same body site (mostly vagina, Additional file 15: Table S3). For example, *Prevotella* contained three PA-OTUs and six NA-OTUs for vagina.

**Fig. 5:**
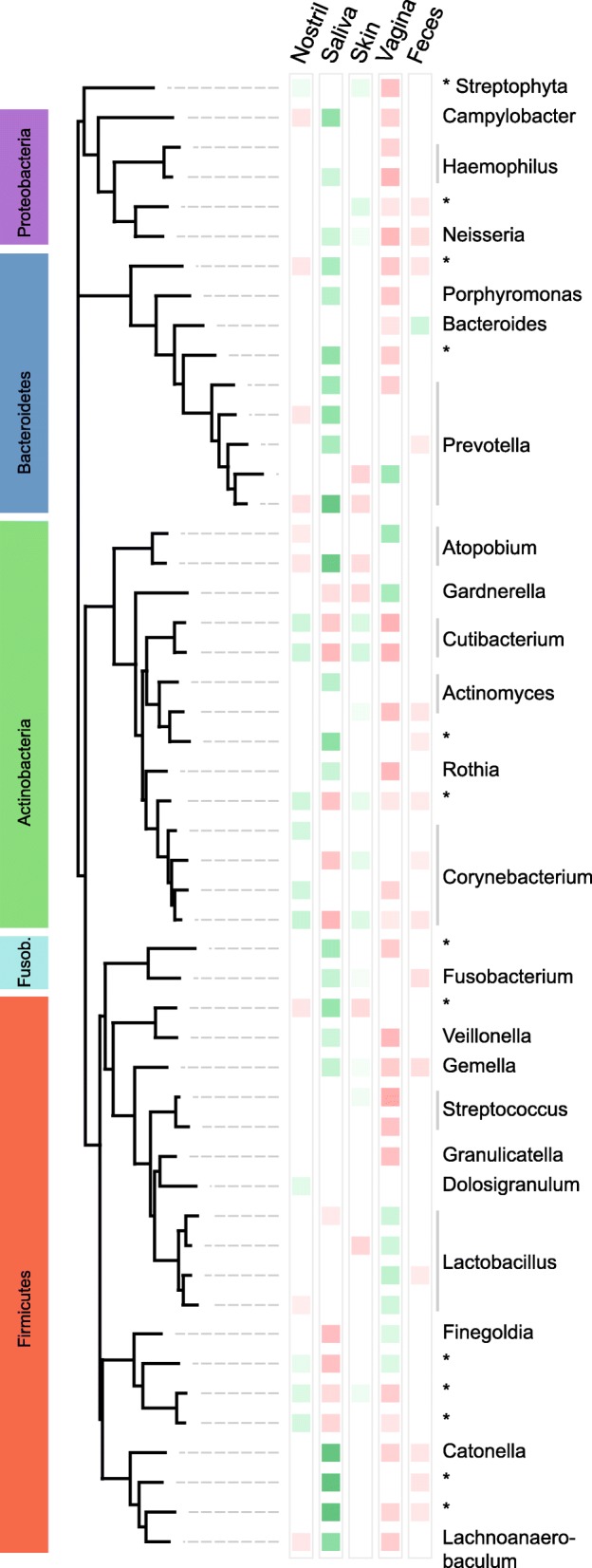
Phylogenetic distribution of the top 50 biomarkers. Biomarker OTUs were chosen based on feature importance (gini impurity) as estimated by RFC-global. Colored blocks represent biomarker association type and strength, measured as normalized mutual information, where green indicates positive and red negative association. OTUs unclassified at the genus level are denoted by “*”. The shortened phylum name stands for *Fusobacteria*

To gain an understanding of the taxonomic relationships among selected biomarkers, we reconstructed a phylogenetic tree for a set of 50 OTUs categorized as the most informative for classification by the Random Forest models (Fig. 5). While most clades at varying tree depths displayed a strong positive association to a single body site, many also included members with shifted habitat preference. For instance, nearly all OTUs in the *Bacteriodetes* clade (phylum level) were positively associated exclusively with saliva. However, one *Prevotella* OTU from this clade was instead positively associated with vaginal sites, and one *Bacteroides* OTU with feces. Similarly, one OTU in the *Atopobium* clade (genus level) was a positive biomarker for saliva while the second one was positively associated with vaginal sites. Overall, association patterns were particularly pronounced for the *Actinobacteria* clade (phylum level): 29% of its members were directly positively or negatively associated to four or more body sites (average associations per OTU, 2.8). In contrast, the *Firmicutes* clade had only 10% OTUs with four or more direct associations (average, 2.1).

Critically, 58% of all biomarker OTUs could not be mapped to any known genus and many of these microbes were among the most important biomarkers (Fig. 5, Additional file 16: Figure S8). Out of this set, 21% could only be confidently classified at the domain or phylum level, with two members surpassing the 90th percentile of feature importance (mean, 40th) (Additional file 16: Figure S8). Furthermore, many of these largely unclassified biomarkers were common: on average, they were found in 1012 samples (up to 4542). In order to characterize the taxonomic neighborhood of biomarkers not confidently mapping to any phylum, we further analyzed their closest 16S rRNA matches and found that most of these OTUs hit *Firmicutes* (55%), *Proteobacteria* (17%), *Bacteroidetes* (11%), and *Tenericutes* (10%), albeit at low sequence identity. Among these phyla, we observed an overrepresentation of unclassified OTUs for *Proteobacteria* (17% unclassified compared to 8% classified) and *Tenericutes* (10% compared to 0.6%).

### Aerobicity is the most defining characteristic of microbial biomarkers found in body sites

To further characterize the selected microbial biomarkers, we collected information on oxygen dependency, shape, gram stain, spore formation, and motility in a literature search and tested which of these microbial traits were enriched among the selected biomarkers at each body site compared to the background of all other biomarkers with the same association type (Table 2). We found the most specific enrichment pattern for PA-OTUs in feces: these microbes were significantly enriched for anaerobes and tended to be rod shaped, spore forming, and motile. In contrast, NA-OTUs for feces tended to be facultative anaerobes or gram positive.

**Table 2:** Enriched physiological traits among biomarker OTUs by body site and association type

Body site	Association type	Physiological trait
Nostril	+	Aerobic, facultative anaerobic, gram positive
	–	No enriched traits
Saliva	+	Spherical shape
	–	Gram positive
Skin	+	Aerobic, facultative anaerobic
	–	Anaerobic
Vagina	+	Facultative anaerobic
	–	No enriched traits
Feces	+	Anaerobic, rod shaped, spore forming, motile
	–	No enriched traits

Across body sites, we found a clear separation by oxygen dependency, where oxygenated body sites (nostril, skin) were enriched for aerobic or facultative anaerobic biomarkers, while feces were enriched for anaerobes. Vagina, being a less oxygenated environment, showed an enrichment of facultative anaerobes only, while the well-oxygenated saliva environment had no significant signal for oxygen dependency.

## Discussion

This study is, to our knowledge, the most comprehensive cross-study evaluation of human body site classification and the first analysis of ecologically informed biomarkers for human body sites. Our results show that aggregating a large number of microbial sequences from diverse studies on human body sites leads to (i) strongly improved classification of body sites and (ii) the identification of parsimonious and likely less biased sets of microbial biomarkers for body sites. In our evaluation of body site classification, we highlight the prediction performance achieved for the detection of mixture components. This characteristic of our classification model is particularly valuable for accuracy-demanding applications as for instance in forensics. Limitations of our study include the observation that the classification of very similar sample types, such as nostril and skin, remains challenging. Moreover, the directly associated microbial biomarkers we report here require further experimental validation.

### Improved classification accuracy in large cross-study datasets

We analyzed a large-scale dataset composed of over 15,000 samples from five human body sites and showed that a RFC model trained on this data (RFC-global) is considerably more accurate for body site prediction than multiple models trained on data from single studies (RFC-single, RFC-single-hmp) used in previous body site classification benchmarks [10, 11, 25]. We found that prediction performance continued improving when including additional studies (after correction for study size), indicating that even more data from undersampled geographic regions or conditions could be useful to further improve the performance of RFC-global. Moreover, we demonstrated that in mixtures comprising microbial communities from two human body sites or a human body site and a soil sample, RFC-global is capable of detecting trace amounts (down to 1% mixture fraction) of a target sample type in many tested cases—this sensitivity was not achieved with RFC-single.

The discrimination power demonstrated here is useful for a number of applications. In a forensic case involving sexual assault, for example discerning whether a stain at a crime scene contains a mixture of vaginal fluid and skin (from different individuals) or whether it contains vaginal fluid and saliva can affect the reconstruction of the crime event. Similarly, the power to discriminate human body site components from soil could help trace body site samples in forensic stains that have been exposed to environmental bacteria for prolonged periods of time, for example in forest environments. Of particular interest is that the high classification performance in mixtures with soil samples was achieved without prior training on soil communities. This inherent robustness of RFC-global to soil-based noise may thus potentially extend to other environments. Since many potential source environments for microbial transfer may still be unknown or undersampled, generic robustness to noise sources would be an important feature. Albeit further confirmation is needed, this also indicates promise for similar classification tasks, like identification of human sewage pollution in water and indoor contamination (or microbial transfer) on hospital surfaces.

As a cautionary note, we observed that classification accuracy and the detection limit in mixtures depended strongly on sample type. For example, sample types harboring relatively similar microbial communities, such as nostril and skin or, to a lesser extent, feces and soil, were harder to distinguish and resulted in more misclassifications and higher detection limits than more distinct sample types, such as nostril and feces. Only high-confidence mixture classifications for similar sample types should thereby be trusted.

### A core set of ecologically informed biomarkers

Applying Generalized Local Learning (GLL) enabled removal of redundant biomarkers whose association was statistically explainable by microbe-microbe associations. This selection step led to a strongly reduced core set of microbial markers that are directly associated with human body sites. We showed that this set precisely captured body site differences, achieving classification accuracy similar to or surpassing the full set of OTUs in unmixed samples. Applying GLL on the Costello et al. dataset [13] identified a reduced set of 12 biomarker OTUs (compared to 635 in the global dataset), resulting in a sharp drop in classification performance (Additional file 10: Figure S6). This drop likely stems from the smaller size and reduced diversity of the Costello et al. dataset, which emphasizes the need for heterogeneous large-scale datasets to fully take advantage of GLL.

We examined two types of biomarkers for each body site: positively associated (PA-OTUs) and negatively associated (NA-OTUs) (Fig. 4b). In contrast to most other body sites, feces were characterized by a larger number of PA-OTUs and few NA-OTUs. This trend was likely a consequence of the distinctness of gut communities from other body sites, as most of these positive, feces-specific biomarkers were identified as negative for at least one other body site (Additional file 12: Figure S7).

Although NA-OTUs are commonly reported by LEfSe [35]—a standard tool for microbial biomarker discovery that does not distinguish between direct and indirect interactions—negative association patterns between microbial taxa and human body sites have to our knowledge not been comprehensively discussed in previous literature. We showed that NA-OTUs are numerous, can achieve levels of accuracy comparable to the use of both OTU types, and result in consistently higher predictive performance than using PA-OTUs alone (Fig. 4d). While NA-OTUs are generally more numerous than PA-OTUs, in particular in nostril, skin, and vagina, this cannot explain observed performance differences, as NA-OTUs also outperform PA-OTUs in body sites with lower NA-OTU proportions. The predictive superiority of NA-OTUs is striking because it indicates that the absence of specific microbial taxa is generally more informative than the presence of usual microbial taxa in an environment. We expect ecological factors driving this strong negative association signal to be mostly non-microbial (e.g., pH, oxygen content, medication), since our GLL analysis reduced the influence of inhibiting ecological microbial associations, such as competition and amensalism. Similarly, positive associations have been corrected for symbiosis and commensalism, leaving unmeasured non-microbial variables as the most likely explanation for observed positive biomarkers. Identifying factors driving the direct associations we report and pinpointing them to particular microbes and body sites would provide important insights into the forces shaping microbial diversity across the human body.

### Taxonomic and phylogenetic patterns of detected biomarkers

The importance of using large aggregated datasets and selecting ecologically informed biomarkers is highlighted in the taxonomic analyses of identified markers. While many biomarker OTUs identified here belonged to previously reported site-specific genera, we also found novel associations. Furthermore, we found that some associations previously reported in single studies were not confirmed in our study. It is worth noting that the conditioning step of GLL can only exclude (but never add) biomarkers; thus, any novel biomarkers found in this study result from the increased size and diversity of the analyzed dataset. Notable examples of novel markers are *Ralstonia* and *Caulobacter* OTUs for skin (Table 1) and an OTU mapping to the archaeal genus *Halovenus* as a biomarker for feces. The latter is surprising because mostly water-dwelling, halophile species of this genus have been described thus far. Since archaeal diversity is underrepresented in current taxonomic databases, it is thus possible that this weakly predictive and low-prevalence OTU belongs to a to-date unidentified archaeal genus able to persist in the human gut.

A number of commonly reported genus associations were not found as direct associations in our analysis. For example, while *Methanobrevibacter* [36] is one of the few archaeal genera consistently identified in the human gut, its known ecological dependence on fermenting bacteria [37] lead to its exclusion as direct biomarker in our study. Similarly, *Veillonella* has been excluded as a biomarker for skin because of its statistical association with a *Streptococcus* OTU, and indeed, symbiosis between *Veillonella* and *Streptococcus* has been previously described [38].

We observed that most major human-associated phyla included both positive and negative biomarkers for all body sites, making high-level taxonomic affiliation only weakly indicative of body site presence. Even at the genus level, we find frequent cases of genera that include biomarker OTUs of the same association type for different body sites. For example, while most *Prevotella* markers were positively associated with saliva, one sub-clade in the phylogenetic tree was associated with vagina (Fig. 5). Furthermore, some genera included PA-OTUs for as many as three distinct body sites, and 5% of all biomarker genera include both PA- and NA-OTUs for the same body site, making these genera highly unreliable for body site classification. We therefore generally recommend analysis at 96% OTU-level resolution or higher to identify predictive biomarkers. A caveat to this approach is that association patterns specific to more general taxonomic levels can be missed. For instance, *Staphylococcus* as a biomarker for skin was not recovered in our analysis because many reads mapping to this genus could not be confidently assigned to one single 96% OTU (see Additional file 17: Text S1). A hybrid approach of unsupervised OTU clusters and supervised taxon assignments may alleviate this problem in future studies. Furthermore, a number of body site-specific strains have recently been described [39], indicating that a strain-level analysis may lead to additional biomarkers and increased classification accuracy in future studies.

Additionally, we note that many microbial biomarkers, some of which are among the most predictive OTUs, could not be precisely taxonomically classified, constituting “microbial dark matter” [40, 41]. For instance, we identified a bacterial OTU distantly related to *Firmicutes* (81% 16S rRNA sequence identity) among the 10% most important biomarkers. It is a strong PA-OTU for saliva and a NA-OTU for nostril, skin, and vagina. Moreover, we observed an overrepresentation of uncharacterized OTUs distantly related to *Proteobacteria* and *Tenericutes*, indicating insufficient coverage of the phylogenetic tree around these phyla in current taxonomic reference databases. We deem it crucial to intensify research on describing uncharacterized human-associated microbes detected in this study in order to elucidate their potential roles in human health and disease.

### Microbial trait enrichment in particular body sites

In terms of physiological traits identified for the microbial biomarkers, we find oxygen dependency to be the most pronounced physiological characteristic among PA and NA biomarkers: aerobic microbes tended to be positively associated with exposed body sites, while (facultative) anaerobic microbes preferred lowly oxygenated sites. Apart from this expected observation, only PA feces biomarkers showed a detailed enrichment pattern for multiple other traits, namely rod shape, motility, and spore formation. Compared to coccoid cells, rod-shaped cells have a higher surface to volume ratio and are therefore more efficient at uptaking substrate [42, 43]. Along the same lines, motility has been found to be an important feature in competitive environments [44, 45]. The enrichment in microbial species with these traits thus indicates that despite being a nutrient-rich environment, the gut selects for microbial traits providing a benefit in competitive habitats. Furthermore, spore-forming lactic acid bacterial species of the *Lactobacillus* genus have been shown to be sensitive or weakly tolerant when exposed to acidic environments and bile acid [46]. However, in endospore form, these bacteria survive the passage through the human stomach and germinate successfully in the gut [47]. The ability to survive the acidic stomach environment that presents a barrier to the gut would therefore confer a clear advantage to microbial species frequently exposed to such environments and suggests that many of these species could be acquired with food, rather than reside permanently in the gut.

### Limitations

Limitations of our study include the use of in silico simulations for mixture analysis. While our protocol uses real samples to create mixtures, it remains to be investigated how mixtures created in a wet lab environment would affect classifier performance. Additionally, we only estimated robustness to environmental noise through soil samples as a typical environmental source. Whether the robustness we observe generalizes to other likely noise sources, such as dust from indoor environments, requires further testing. Importantly, a classifier trained only on GLL-selected biomarkers was markedly less robust to environmental noise, likely because no markers were selected to discriminate this signal. It is therefore crucial to use the classifier trained on all OTUs if environmental mixtures are expected. Finally, the direct microbe-environment associations we report here are based on statistical relationships and whether these reflect real direct environmental dependencies requires experimental validation.

## Conclusions

The present study is part of a recent development, where the ongoing growth and diversification of microbial sequencing studies of human body sites, coupled with adequate statistical techniques to mine emerging patterns from this data, catalyzes discovery. We are confident that this data-intense approach will continue to expand our understanding of the human microbiome and lead to generalized insights into our microbial ecosphere.

## Methods

### Data acquisition

We first selected a set of representative body sites: saliva, skin, vagina, and feces. We further included the body site nostril due to its known similarity with skin, in order to also test classification accuracy on the more difficult distinction between skin and nostril in later analysis steps.

Studies from the NCBI Sequence Read Archive database (SRA, [29]) were filtered for human samples through automated parsing of annotation keywords, matching at least one of the following rules: (1) “Human” or “*Homo sapiens*” is found in the host name field, (2) “9606” is found in either the host taxon ID or sample taxon ID field, or (3) the pattern “human <*> metagenome” is found in the organism field, where “<*>” is a wildcard for a single word (e.g., “gut”) or empty. On these filtered human samples, we then conducted a second body site-targeted search with the keywords “saliva,” “tongue,” “nostril,” “nares,” “vagina,” “fornix,” “retroauricular crease,” “antecubital fossa,” “skin,” and “feces.” Random subsets of samples for each body site were manually checked via the SRA web service to verify that the filtered samples were of human origin and belonged to the habitats assigned by the automated pipeline. In case of mismatches, samples were removed from the pool. Similarly, soil samples were retrieved from the SRA through the keyword “soil” and subsequently filtered for samples with no body site-related keyword annotations.

### OTU mapping, taxonomic classification, and filtering

Raw sequence data for 50,273 sequenced samples (independent sequencing runs of biological samples) from the keyword-filtered studies was downloaded from the NCBI SRA database. Reads were quality filtered using custom programs that trimmed reads to the first two consecutive low-quality bases (≤ 10) and discarded reads smaller than 75 bp or having a fraction of low-quality reads larger than 5%. MAPseq v1.0 [30] was used to map the filtered reads to the reference of full-length 16S/18S rRNA sequences provided with MAPseq which includes representatives for 61,899 OTUs at the 96% identity cutoff. The results of MAPseq were parsed and an OTU count table was created using the assignments to OTUs at 96% sequence identity with a minimum confidence of 0.5. Taxonomy was assigned to OTUs based on a 90% consensus over the full taxonomic lineages of all OTU member sequences. For sequences belonging to RefSeq [48] genomes or culture collection strains, the annotated taxonomy as provided by NCBI in December 2017 was used. Other sequences were taxonomically classified through mapping onto the RefSeq set using MAPseq and a confidence threshold of 0.5.

An initial analysis (see Additional file 17: Text S1) showed that samples with fewer than 20 unique OTUs tended to be noisy; we therefore excluded these from the analysis. We then computed the normalized mutual information between OTUs and performed hierarchical clustering (complete linkage, Euclidean distance) to group highly similar OTUs with normalized mutual information higher than 0.9 together, as these OTUs were hard to distinguish from each other by GLL, the biomarker discovery algorithm we applied. For each group, a random representative was chosen. After all filtering steps, a subset of 15,082 samples remained for analysis.

### Classification of body sites

Counts in the OTU table were normalized by the total number of mapped reads per sample, resulting in relative abundances. Next, the dataset was split into five distinct training and validation sets, retaining the proportions of samples across body sites constant for each subset (stratified k-fold split). For each subset, a Random Forest Classifier (RFC, [15]) was trained with the python package scikit-learn [49]. Hyper parameters were optimized based on a grid search across a stratified inner fourfold cross-validation on each respective training set. Possible parameter values were as follows: number of trees 500, 1000, or 2000; maximum number of features $\frac{1}{2}\cdotp \sqrt{\mathrm{features}}$,$\sqrt{\mathrm{features}}$, $2\times \sqrt{\mathrm{features}}$. Class weights were automatically adjusted to cope with class imbalance, and the optimization objective for the inner cross-validation loop was the F1 score. Classifier output probabilities were additionally calibrated through a non-parametric procedure based on isotonic regression [50] implemented in scikit-learn. All five classifiers were evaluated on their respective validation sets to estimate the generalization error of the final classifier trained on the whole dataset (RFC-global).

For RFC-single and RFC-single-hmp, the same procedure was slightly adapted to use only samples from the study by Costello et al. [13] or the Human Microbiome Project [26] for training. Furthermore, vaginal samples were excluded from the validation sets for the comparison between RFC-global and RFC-single since samples of this type were not collected in [13].

For “weighted” F1 score analyses, we weighted each sample by the inverse of the number of samples belonging to its associated study. These weights were then passed as “sample_weight” parameter in the scikit-learn F1 score function.

Biased validation and training sets were created by keeping all samples from one body site (*B*_bias_) and then randomly down-sampling all other body sites until each had 10% of the sample count of *B*_bias_.

### Mixture simulations

Artificial sample mixtures from two body sites *B*_target_ and *B*_background_ were created through the following procedure: (i) randomly choose one sample taken from *B*_target_ and *B*_background_ respectively; (ii) compute relative frequencies of each OTU in these samples and use the frequencies as base probabilities of OTU-drawing; (iii) weight the probabilities according to the desired mixture fraction *F*_background_, which determines how similar the mixed sample should be to the sample from *B*_background_; (iv) randomly choose OTU sequences based on these weighted probabilities until *n* sequences were chosen, where *n* is the weighted average of sequences in the two samples, with weights *F*_background_ and 1 − *F*_background_. The procedure was repeated for each pair of body sites along a gradient of increasing mixture fractions.

To estimate performance, the data was split into the same training and validation sets as described previously for the classification of unmixed samples. For each of these splits, the classifiers previously trained on the respective training sets were used, while validation sets were further processed, separately for each body site pair *B*_target_ and *B*_background_. For this processing, samples in the validation sets were first reduced to only samples from *B*_target_ and *B*_background_, followed by in silico mixture of all *B*_target_ validation samples with randomly picked *B*_background_ validation samples, using increasing mixture fractions *F*_background_. AUC scores were finally computed for each mixed validation set and pre-trained classifier.

We also determined thresholds for the correct identification of a target body site in a mixture. To do so, we first prepared in silico mixtures as described above for all training samples. Then we computed a precision-recall curve for these mixed training samples and picked the threshold that yielded the optimal F1 score on that curve. Subsequently, F1 scores were used to quantify the threshold-adjusted prediction performance of our Random Forest models on mixed test sets (see previous paragraph). This procedure was repeated for each combination of body sites and mixture fractions, leading to a threshold table with 5 × 4 × 10 = 200 entries. For this analysis, only thresholds were adjusted; the classifier decision trees and calibrator were not trained on mixed samples.

### Identification of microbial biomarkers

While Random Forest Classifiers perform intrinsic feature selection which can yield insights into which OTUs the classifier estimates to be most predictive [11, 12, 15], this approach has a number of shortcomings. Firstly, if features are highly correlated, the classifier tends to arbitrarily pick one of them and discard the others, leading to the removal of potentially biologically interesting OTUs. Since microbes interact with each other and live in complex ecological networks of mutual dependencies, such correlations are inevitable. Furthermore, deciding on a cutoff for how many OTUs to label as biomarkers based on feature importance (gini impurity in our case) can be difficult.

To identify the core set of microbial markers directly associated with a body site, we applied Generalized Local Learning (GLL, [27]) to address these shortcomings. The approach detects OTUs whose association with a habitat cannot be statistically explained by their relationship with other microbes, effectively exploiting ecological dependencies among OTUs to make biomarker discovery more parsimonious. Furthermore, it internally uses statistical tests of independence, which apply well-studied significance cutoffs, and avoids classifier-specific inductive biases [31].

GLL was instantiated with semi-interleavedHITON-PC as edge-finding algorithm and mutual information as test metric (proportional to the classic *G*-test, [51]). We ran the algorithm with the following parameters: max-k = 3, h-ps = 5, and alpha = 0.05. Prior to biomarker discovery, OTU abundances in the OTU table were binarized, where an OTU was assigned the value 1 if at least one sample read mapped to the OTU and 0 if not, in order to allow discretized mutual information tests and reduce sequencing depth biases. We ran GLL separately for each body site, using a custom implementation in the python [52] and cython [53] programming languages. False discovery rate adjustment of *p* values [54] was applied prior to the GLL conditioning step. Whether an OTU was positively or negatively associated with a body site was estimated by the sign of the Spearman correlation coefficient between binarized OTU and body site. We found identical association type assignments for odds ratios and linear discriminant analysis effect sizes.

### Phylogenetic analysis of biomarkers

The biomarkers identified by GLL were weighted based on feature importance (gini impurity) inferred by RFC-global, and the top 50 most important biomarkers as estimated by feature importance (gini impurity) were chosen for phylogenetic analysis. A multiple sequence alignment for the selected biomarkers was extracted as a subset of the publicly available alignment of all OTUs in the reference database, created with INFERNAL version 1.1.2 [55] and microbial secondary structure model SSU-ALIGN [56]. Based on this alignment, a phylogenetic tree was reconstructed using fasttree version 2.1.3 [57] using the GTR substitution model and otherwise default options.

### Collection of microbial trait information

Across all PA and NA biomarker OTUs, we created a list of genera and reviewed primary literature and the public database MicrobeWiki [58] to assign a list of phenotypic characteristics to them. Major categories were aerobicity (subcategories: aerobe, anaerobe, facultative anaerobe), gram stain (gram positive, gram negative), cell shape (rod, spherical, helical), spore formation (forms spores, does not form spores), and motility (motile, non-motile). When no information was found, the trait was labeled as missing, while if more than one sub category was described by different sources, all alternatives were kept and used later for the statistical analysis. This trait information was then extrapolated to all OTUs with mapped genus information.

### Statistical analysis of microbial traits

For each marker OTU subset *S* from each combination of body site and association type, as well as each trait sub-category (e.g., anaerobic), we tested whether the sub-category was significantly enriched within *S* compared to the background of marker OTUs with the same association as *S*, but associated to a different body site. To this end, we conducted Fisher’s exact test (alpha 0.05, one-tailed), followed by false discovery rate adjustment of *p* values [54].

## Additional files

Additional file 1: **Table S4. **Accessions for sequencing runs, sequencing samples, and projects contained in GlobalBodysites. (TSV 440 kb)

Additional file 2: **Figure S10**. Additional performance comparisons of RFC-global. (A) Comparison of RFC-global to a classifier trained on the Human Microbiome Project subset of GlobalBodysites (RFC-single-hmp). “unweighted”: default F1 scores are computed, without applying weights; “weighted”: samples are weighted inversely to the size of the study they belong to for F1 score calculation, resulting in a penalty for large studies and higher importance of smaller studies. (B) Robustness of RFC-global and two of its variations to body site proportion biases in the validation sets. Test set definitions are as follows: “*-biased”, body site “*” had 10× more samples in the validation set than other body sites; “Equal”, all body sites had equal proportions (equivalent to the site with the fewest samples). Classifiers are: “RFC-global”, the original RFC-global classifier; “RFC-global-balanced”, trained on equal body site proportions; “RFC-global-Feces-biased”, trained on a biased set with 10× more feces samples than other body sites. (PDF 426 kb)

Additional file 3: **Figure S1.** Comparison of OTU importance between RFC-global and RFC-single. (A) Overlap of predictive OTUs (feature importance > 0) between classifiers, (B) joint distribution of unpredictive (U.P.) and predictive (P.) OTUs, and (C) direct comparison of feature importances for 1277 OTUs predictive in both classifiers. (PDF 626 kb)

Additional file 4: **Figure S11.** Improvement of RFC-global performance with increasing numbers of studies and samples. “unweighted”: default F1 scores are computed without applying weights; “weighted”: samples are weighted inversely to the size of the study they belong to for F1 score calculation, resulting in a penalty for large studies and higher importance of smaller studies. (A) Performance in relation to increasing numbers of studies, starting with only the HMP dataset. (B) Re-mapping of (A) to the numbers of samples included in each set of studies. (PDF 413 kb)

Additional file 5: **Figure S2.** Discrimination performance of RFC-global on mixed samples. Along a gradient of increasing mixture fractions (0 to 100%), unseen samples for all pairs of body sites were combined into mixed samples using an in silico procedure (see the “Methods” section) and then predicted by RFC-global. Prediction performance was quantified in terms of AUC. (PDF 508 kb)

Additional file 6: **Figure S4.** Comparison of discrimination performance between RFC-global and RFC-single on mixed samples. Along a gradient of increasing mixture fractions (0 to 100%), unseen samples for all pairs of body sites were combined into mixed samples using an in silico procedure (see the “Methods” section) and then predicted by both classifiers. Prediction performance was quantified in terms of AUC. (PDF 456 kb)

Additional file 7: **Figure S3.** Discrimination performance of RFC-global on mixed samples. Along a gradient of increasing mixture fractions (0 to 100%), unseen samples for all pairs of body sites were combined into mixed samples using an in silico procedure (see the “Methods” section) and then predicted by RFC-global (thresholds optimized on the training sets). Prediction performance was quantified in terms of F1 score. (PDF 501 kb)

Additional file 8: **Figure S5.** Comparison of discrimination performance between RFC-global with all OTUs and only biomarker OTUs on samples contaminated with soil. Along a gradient of increasing mixture fractions (0 to 100%), unseen samples of each body site were contaminated with soil using an in silico procedure (see the “Methods” section) and then predicted by RFC-global, using each respective OTU set. Prediction performance was quantified in terms of AUC. (PDF 375 kb)

Additional file 9: **Table S5. **Comprehensive information of all biomarker OTUs, including marker association types, marker strength, and meta-information such as NCBI 16S accessions and taxonomic lineages. (TSV 68 kb)

Additional file 10: **Figure S6.** Prediction performance across body sites for RFC-global vs. RFC-single, as well as all OTUs vs. only biomarker OTUs. Ecologically informed biomarker OTUs were extracted from the whole GlobalBodysites dataset for RFC-global and from a single-study subset [13] for RFC-single. Prediction performance was measured as (A) AUC and (B) F1 score. (PDF 389 kb)

Additional file 11: **Figure S9.** Comparison of prevalence and mean relative abundance between NA-OTUs, PA-OTUs (strictly positive, PA; strictly negative, NA), and non-biomarkers. Mean relative abundances were scaled by taking their square root. (A) Sample quantities calculated across all samples. (B) Sample quantities stratified by body site, where PA- and NA-OTUs only include biomarkers for each respective body site. (PDF 3389 kb)

Additional file 12: **Figure S7.** Overlaps of identified biomarker OTUs between body sites. Diagonal: distribution of PA-OTUs and NA-OTUs for each body site. Upper triangle: pairwise overlaps of biomarker OTUs for each body site, quantified by Jaccard similarity (indicated by “Jacc”). Lower triangle: normalized joint distribution of PA-OTUs and NA-OTUs for each body site pair. High values indicate large fractions of OTUs with one association type in the first row body site (rows) and a second association type in the second body site (columns). (PDF 816 kb)

Additional file 13: **Table S1.** Top 5 most important positively associated biomarker genera per body site, measured by maximum feature importance in RFC-global among biomarker OTUs of each genus. (DOCX 7 kb)

Additional file 14: **Table S2**. Novel and previously described positive associations between genera and body sites. (DOCX 23 kb)

Additional file 15: **Table S3**. Numbers of NA- and PA-OTUs per genus and body site. (TSV 5 kb)

Additional file 16: **Figure S8.** Feature importances by taxonomic classification quality. For each taxonomic rank, shows feature importances of all biomarker OTUs confidently classified down to that rank, but not further. (PDF 343 kb)

Additional file 17: **Text S1**. Supplementary methods and analyses. (DOCX 21 kb)

## Abbreviations

AUC: Area under the ROC curve
GLL: Generalized Local Learning
NA-OTU: Negatively associated biomarker OTU
OTU: Operational taxonomic unit
PA-OTU: Positively associated biomarker OTU
RFC: Random Forest Classifier
ROC: Receiver operating characteristic
SRA: Sequence Read Archive
WGS: Whole genome sequencing
