# Supplementary figures and images for: Ecologically informed microbial biomarkers and accurate classification of mixed and unmixed samples in an extensive cross-study of human body sites
Source: Microbiome. 2018 Oct 24;6:192. doi: 10.1186/s40168-018-0565-6 (PMC6201589; doi:10.1186/s40168-018-0565-6)

**a**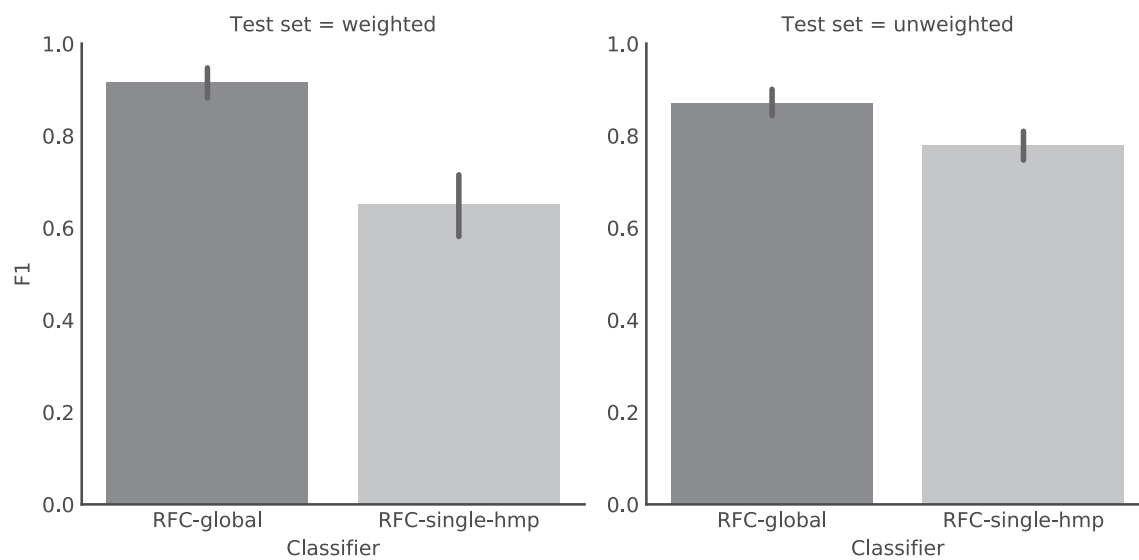**b**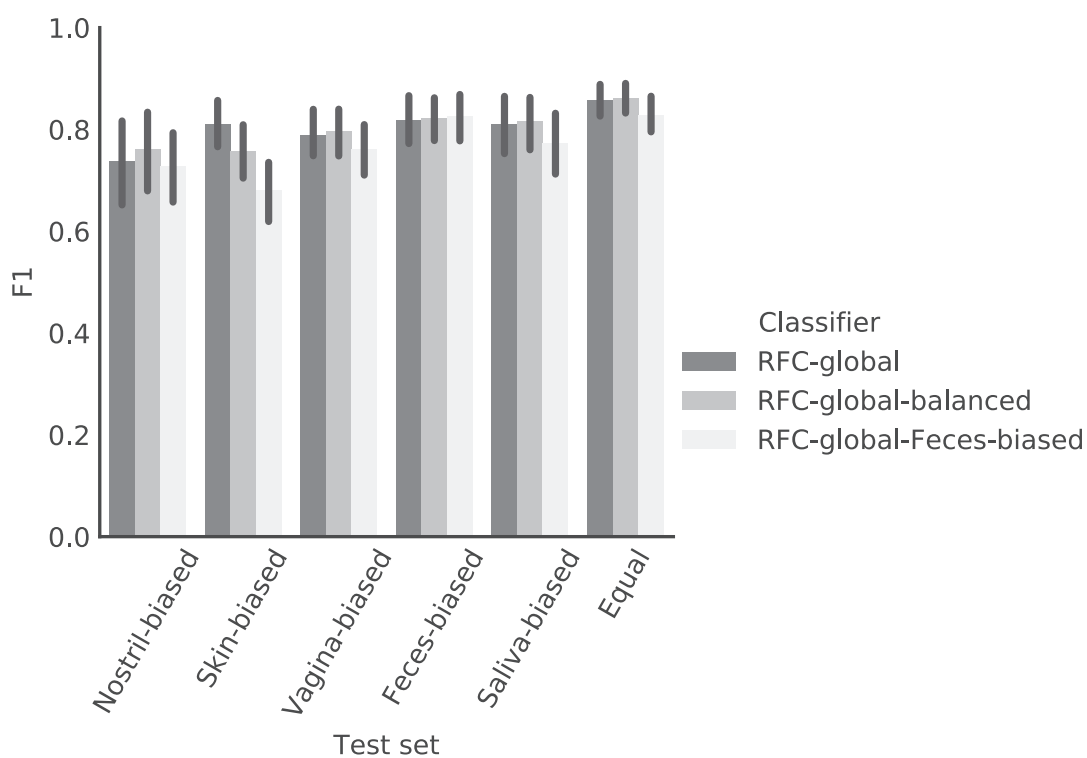**c**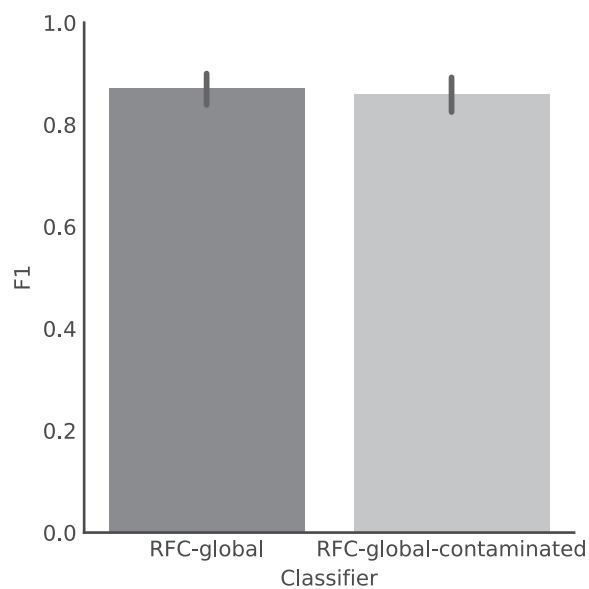

Supplement: Supplementary file 2 — Figure S10. Additional performance comparisons of RFC-global. (A) Comparison of RFC-global to a classifier trained on the Human Microbiome Project subset of GlobalBodysites (RFC-single-hmp). “unweighted”: default F1 scores are computed, without applying weights; “weighted”: samples are weighted inversely to the size of the study they belong to for F1 score calculation, resulting in a penalty for large studies and higher importance of smaller studies. (B) Robustness of RFC-global and two of its variations to body site proportion biases in the validation sets. Test set definitions are as follows: “*-biased”, body site “*” had 10× more samples in the validation set than other body sites; “Equal”, all body sites had equal proportions (equivalent to the site with the fewest samples). Classifiers are: “RFC-global”, the original RFC-global classifier; “RFC-global-balanced”, trained on equal body site proportions; “RFC-global-Feces-biased”, trained on a biased set with 10× more feces samples than other body sites. (PDF 426 kb) [file 40168_2018_565_MOESM2_ESM.pdf]

**a**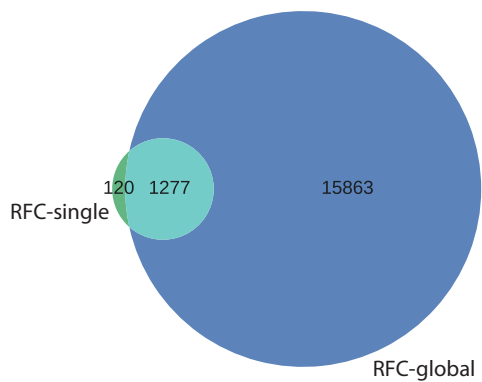**b**

|            |       | RFC-global |        |        |
|------------|-------|------------|--------|--------|
|            |       | U.P.       | P.     | Total  |
| RFC-single | U.P.  | 17'017     | 15'863 | 32'881 |
|            | P.    | 120        | 1'277  | 1'397  |
|            | Total | 17'138     | 17'140 | 34'278 |

**c**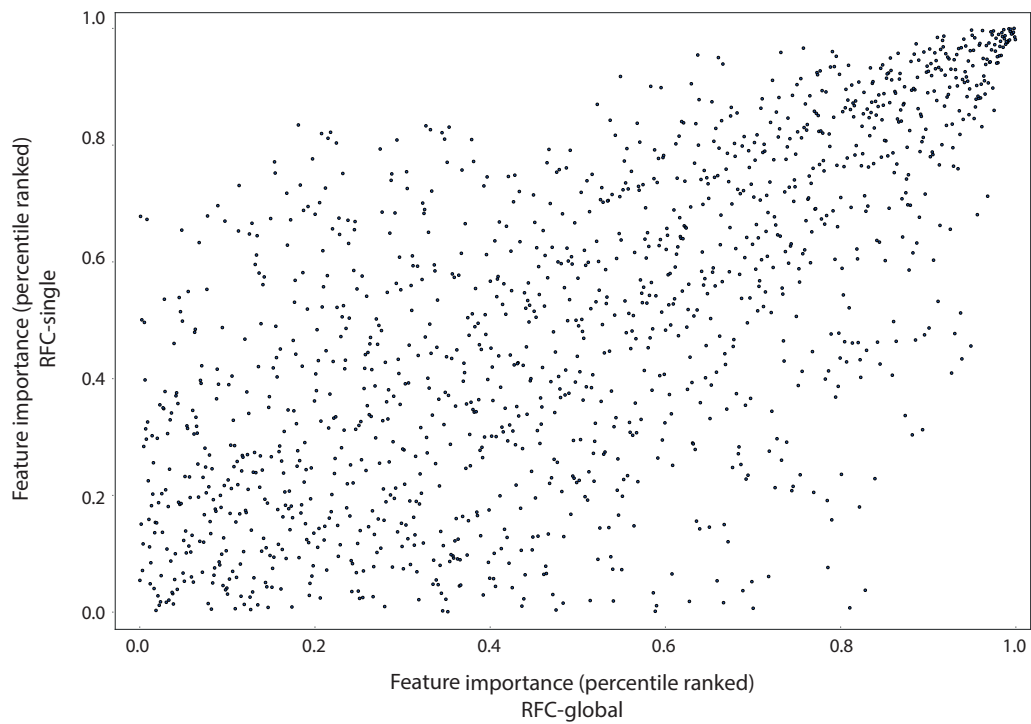

Supplement: Supplementary file 3 — Figure S1. Comparison of OTU importance between RFC-global and RFC-single. (A) Overlap of predictive OTUs (feature importance > 0) between classifiers, (B) joint distribution of unpredictive (U.P.) and predictive (P.) OTUs, and (C) direct comparison of feature importances for 1277 OTUs predictive in both classifiers. (PDF 626 kb) [file 40168_2018_565_MOESM3_ESM.pdf]

**a**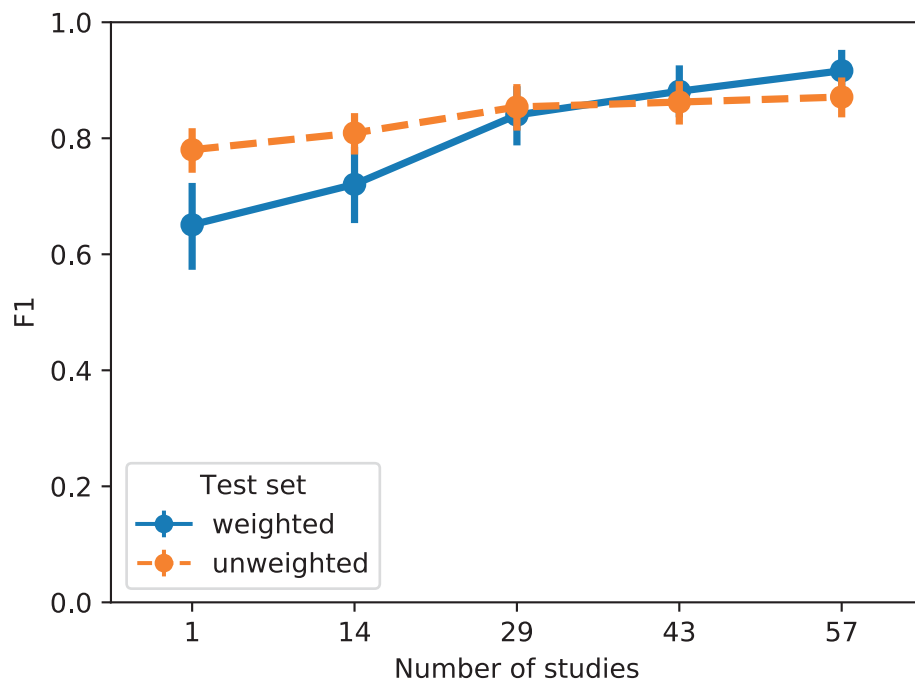**b**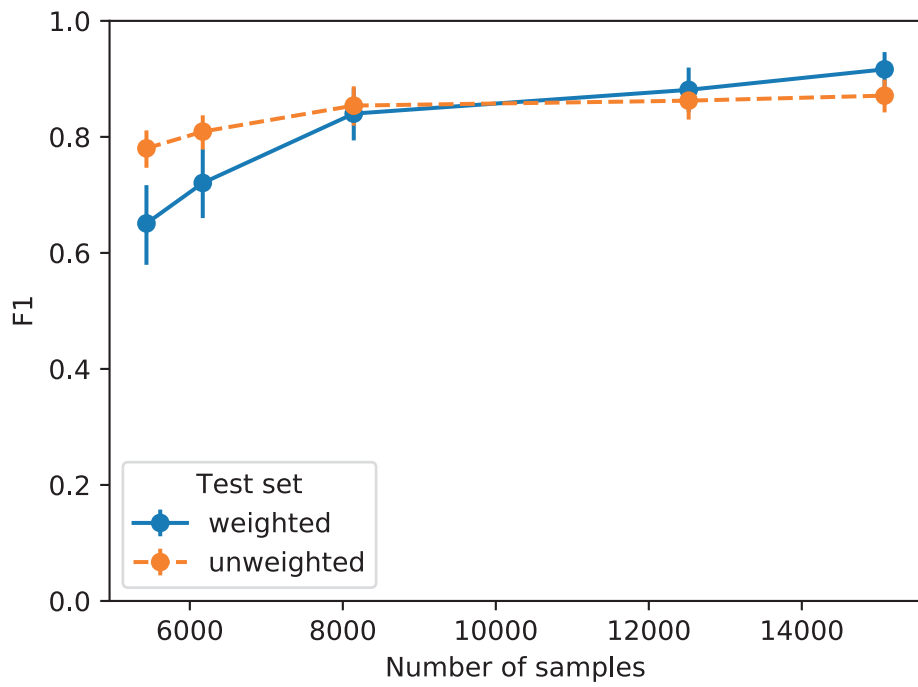

Supplement: Supplementary file 4 — Figure S11. Improvement of RFC-global performance with increasing numbers of studies and samples. “unweighted”: default F1 scores are computed without applying weights; “weighted”: samples are weighted inversely to the size of the study they belong to for F1 score calculation, resulting in a penalty for large studies and higher importance of smaller studies. (A) Performance in relation to increasing numbers of studies, starting with only the HMP dataset. (B) Re-mapping of (A) to the numbers of samples included in each set of studies. (PDF 413 kb) [file 40168_2018_565_MOESM4_ESM.pdf]

Target body site

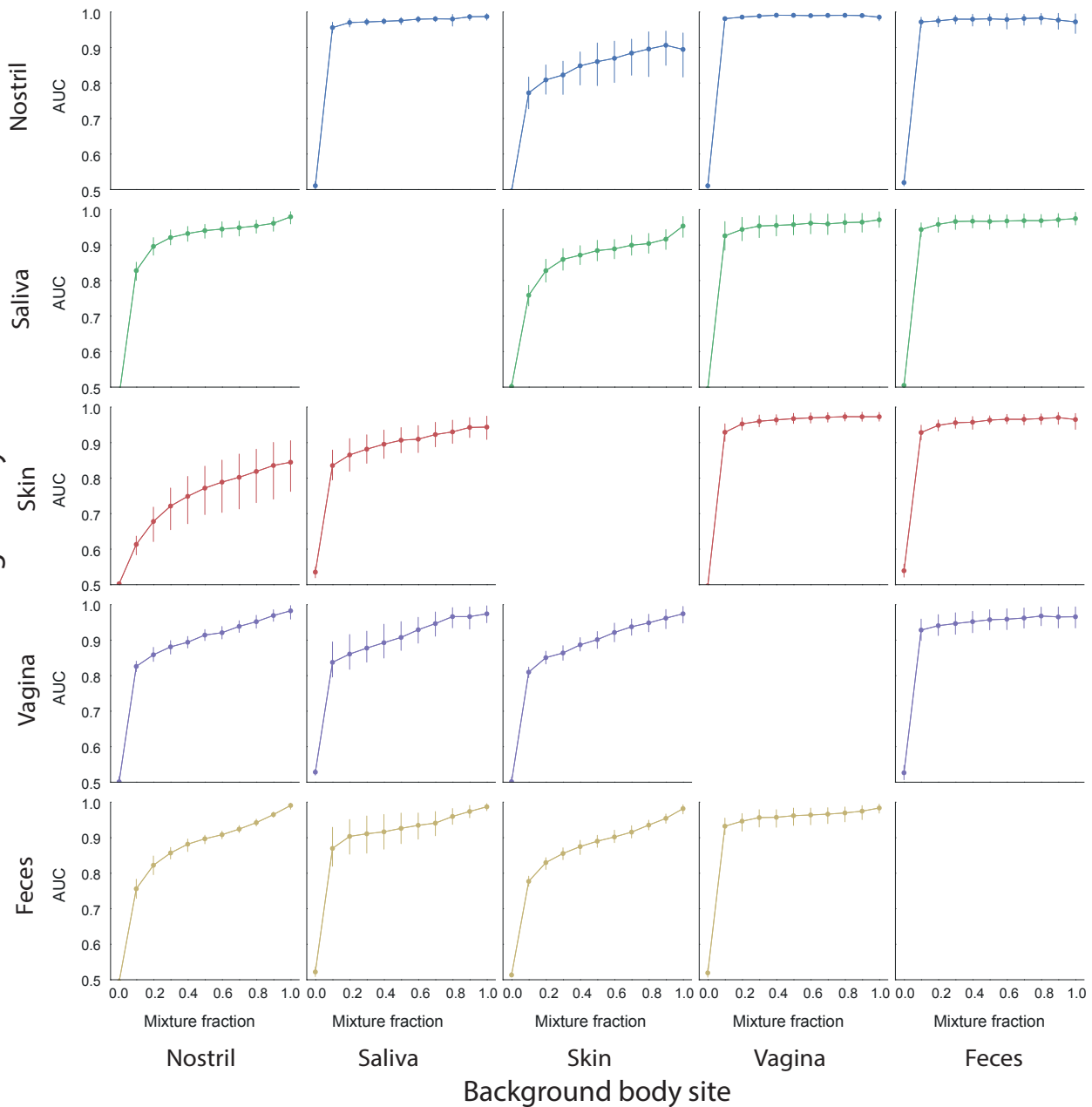

Supplement: Supplementary file 5 — Figure S2. Discrimination performance of RFC-global on mixed samples. Along a gradient of increasing mixture fractions (0 to 100%), unseen samples for all pairs of body sites were combined into mixed samples using an in silico procedure (see the “Methods” section) and then predicted by RFC-global. Prediction performance was quantified in terms of AUC. (PDF 508 kb) [file 40168_2018_565_MOESM5_ESM.pdf]

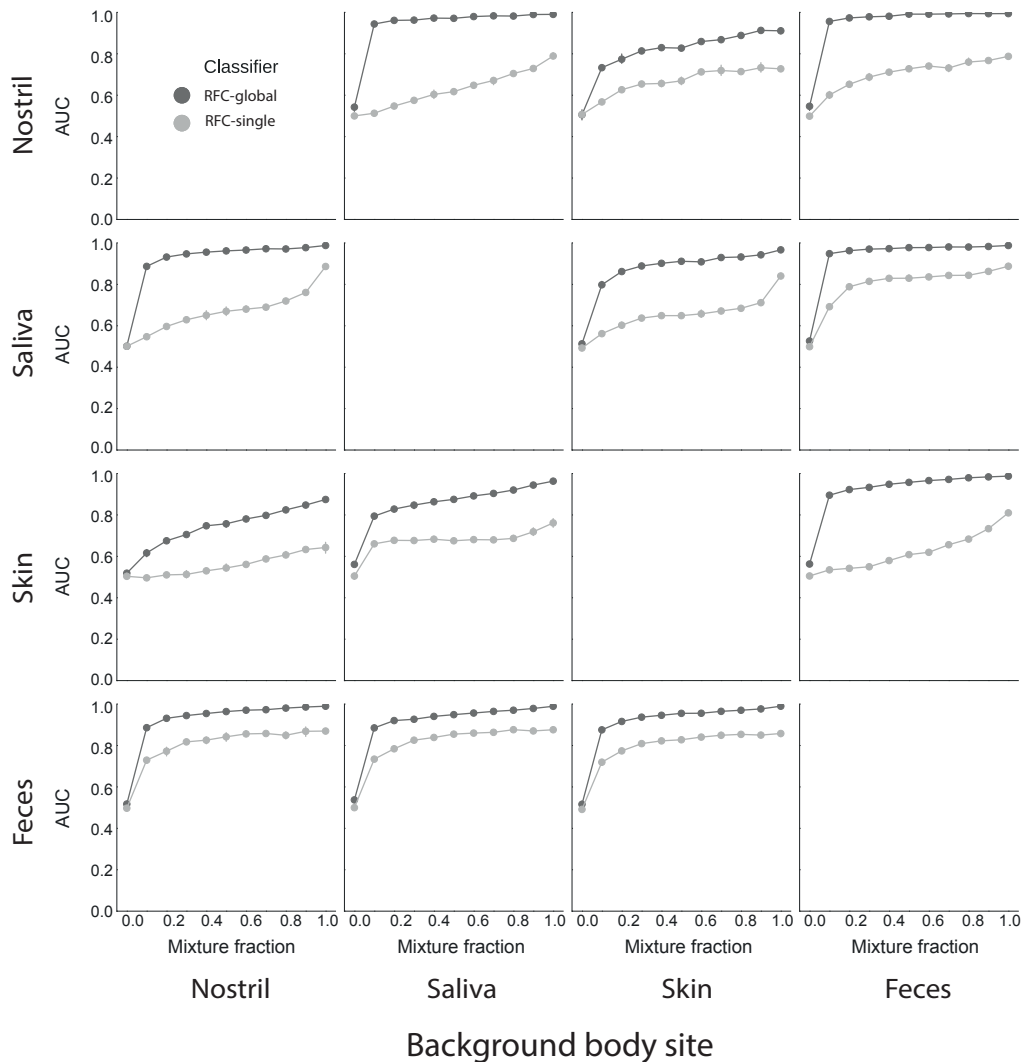

Supplement: Supplementary file 6 — Figure S4. Comparison of discrimination performance between RFC-global and RFC-single on mixed samples. Along a gradient of increasing mixture fractions (0 to 100%), unseen samples for all pairs of body sites were combined into mixed samples using an in silico procedure (see the “Methods” section) and then predicted by both classifiers. Prediction performance was quantified in terms of AUC. (PDF 456 kb) [file 40168_2018_565_MOESM6_ESM.pdf]

Target body site

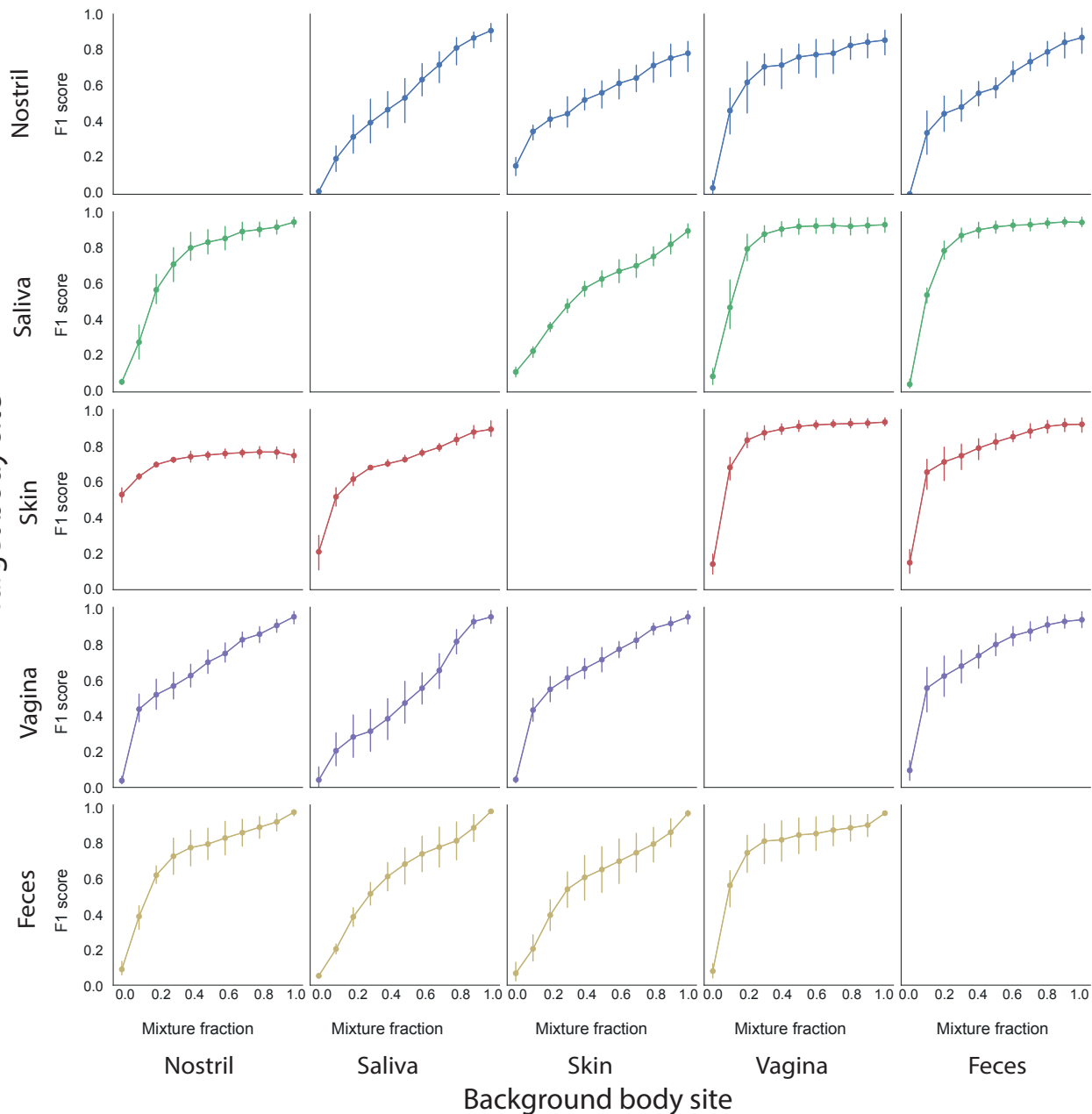

Supplement: Supplementary file 7 — Figure S3. Discrimination performance of RFC-global on mixed samples. Along a gradient of increasing mixture fractions (0 to 100%), unseen samples for all pairs of body sites were combined into mixed samples using an in silico procedure (see the “Methods” section) and then predicted by RFC-global (thresholds optimized on the training sets). Prediction performance was quantified in terms of F1 score. (PDF 501 kb) [file 40168_2018_565_MOESM7_ESM.pdf]

**a**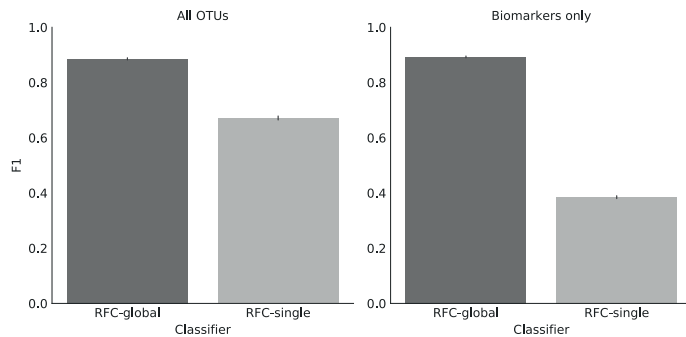**b**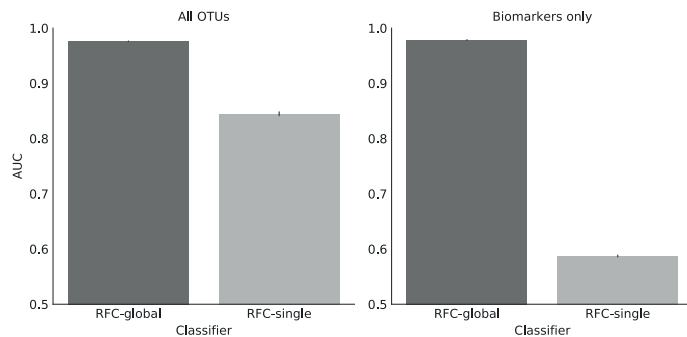

Supplement: Supplementary file 10 — Figure S6. Prediction performance across body sites for RFC-global vs. RFC-single, as well as all OTUs vs. only biomarker OTUs. Ecologically informed biomarker OTUs were extracted from the whole GlobalBodysites dataset for RFC-global and from a single-study subset [13] for RFC-single. Prediction performance was measured as (A) AUC and (B) F1 score. (PDF 389 kb) [file 40168_2018_565_MOESM10_ESM.pdf]

**a**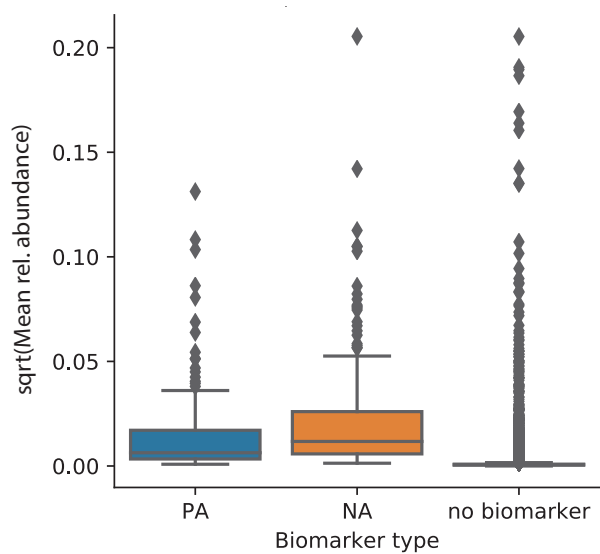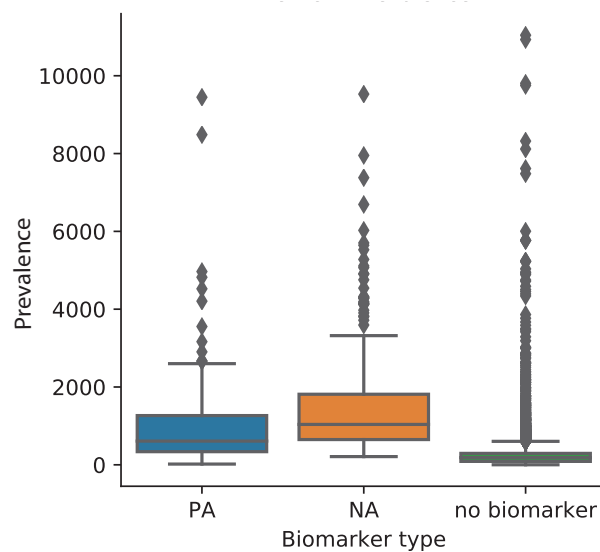**b**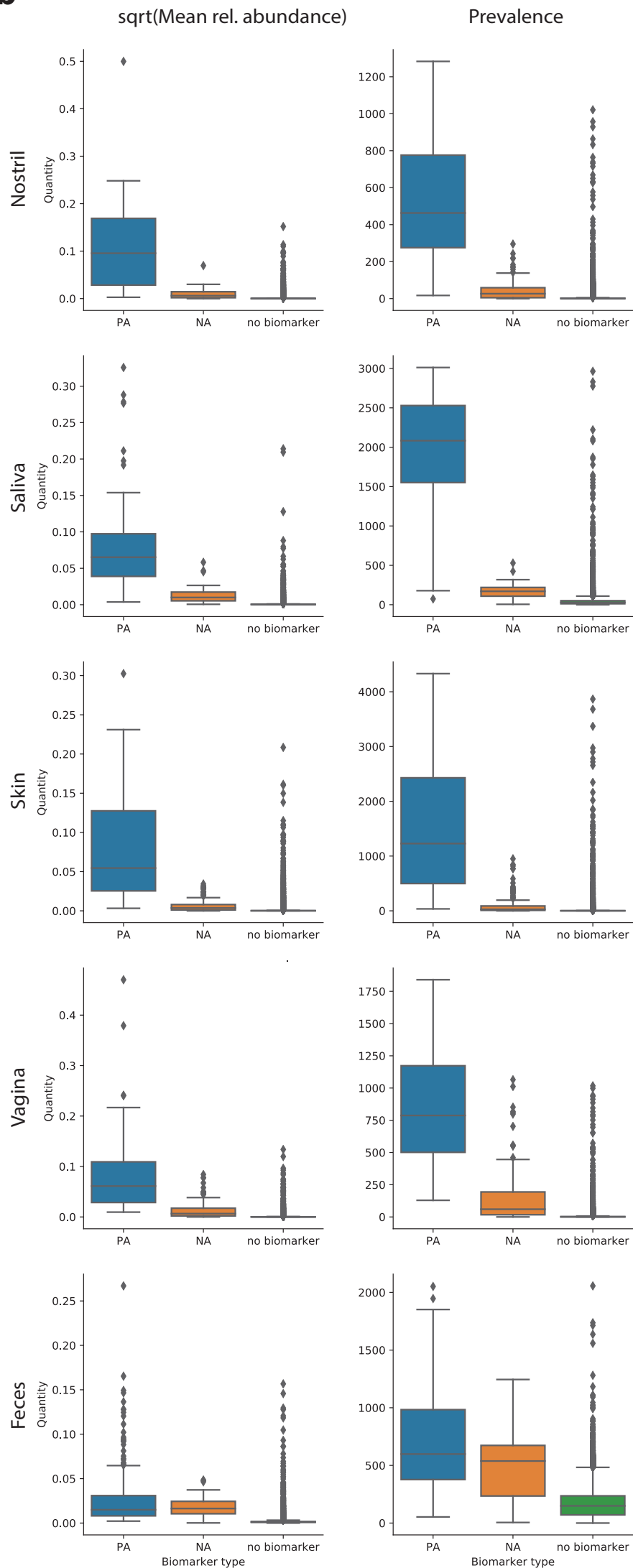

Supplement: Supplementary file 11 — Figure S9. Comparison of prevalence and mean relative abundance between NA-OTUs, PA-OTUs (strictly positive, PA; strictly negative, NA), and non-biomarkers. Mean relative abundances were scaled by taking their square root. (A) Sample quantities calculated across all samples. (B) Sample quantities stratified by body site, where PA- and NA-OTUs only include biomarkers for each respective body site. (PDF 3389 kb) [file 40168_2018_565_MOESM11_ESM.pdf]

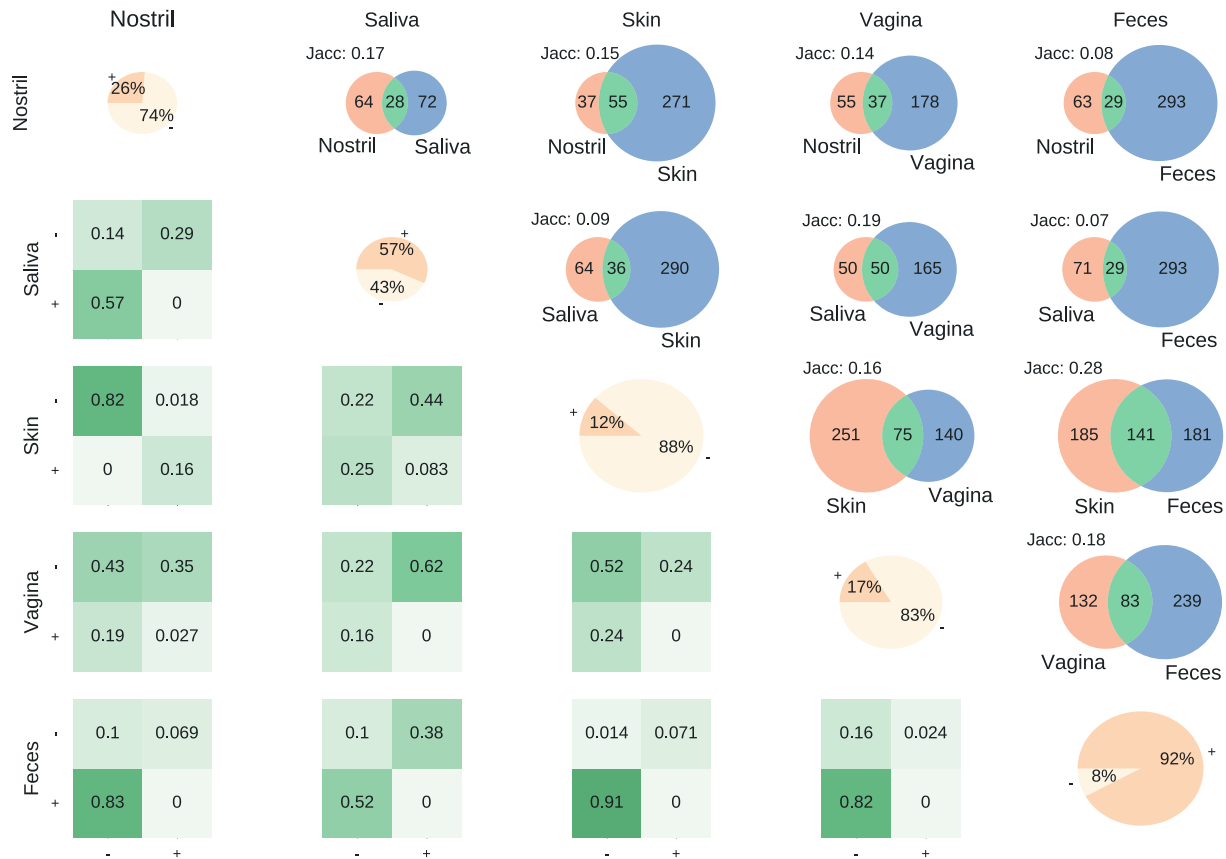

Supplement: Supplementary file 12 — Figure S7. Overlaps of identified biomarker OTUs between body sites. Diagonal: distribution of PA-OTUs and NA-OTUs for each body site. Upper triangle: pairwise overlaps of biomarker OTUs for each body site, quantified by Jaccard similarity (indicated by “Jacc”). Lower triangle: normalized joint distribution of PA-OTUs and NA-OTUs for each body site pair. High values indicate large fractions of OTUs with one association type in the first row body site (rows) and a second association type in the second body site (columns). (PDF 816 kb) [file 40168_2018_565_MOESM12_ESM.pdf]

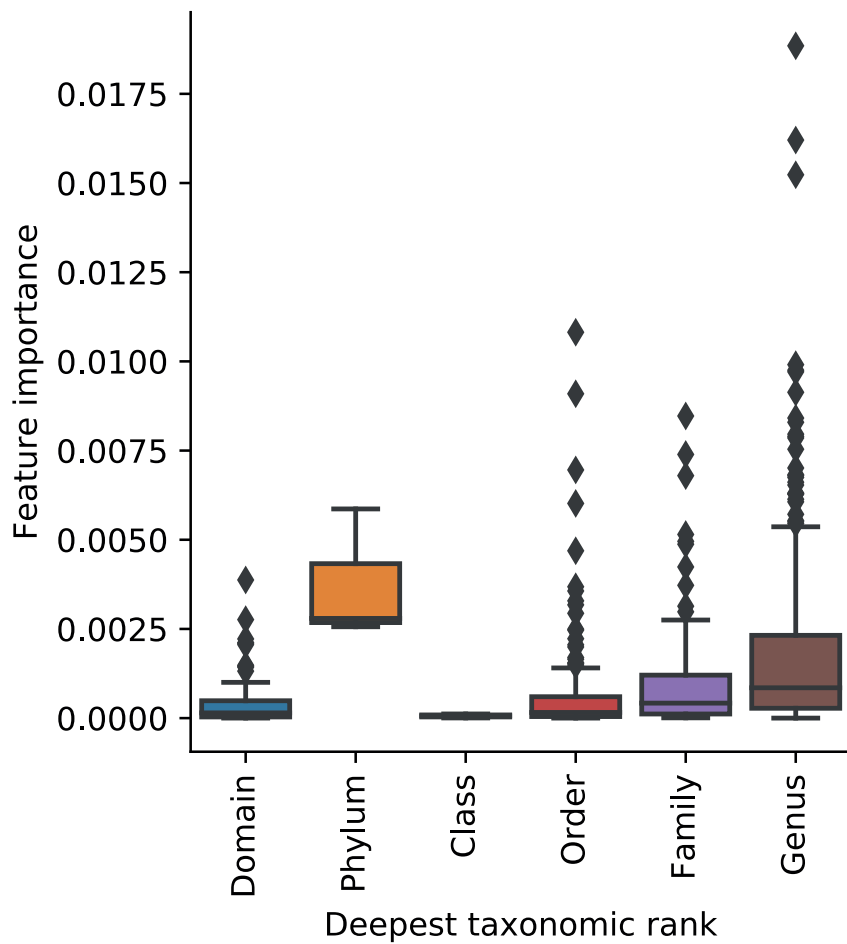

Supplement: Supplementary file 16 — Figure S8. Feature importances by taxonomic classification quality. For each taxonomic rank, shows feature importances of all biomarker OTUs confidently classified down to that rank, but not further. (PDF 343 kb) [file 40168_2018_565_MOESM16_ESM.pdf]
