# Supplementary material for: Ecologically informed microbial biomarkers and accurate classification of mixed and unmixed samples in an extensive cross-study of human body sites
Source: Microbiome. 2018 Oct 24;6:192. doi: 10.1186/s40168-018-0565-6 (PMC6201589; doi:10.1186/s40168-018-0565-6)
Supplement: Supplementary file 8 — Figure S5. Comparison of discrimination performance between RFC-global with all OTUs and only biomarker OTUs on samples contaminated with soil. Along a gradient of increasing mixture fractions (0 to 100%), unseen samples of each body site were contaminated with soil using an in silico procedure (see the “Methods” section) and then predicted by RFC-global, using each respective OTU set. Prediction performance was quantified in terms of AUC. (PDF 375 kb) [file 40168_2018_565_MOESM8_ESM.pdf]

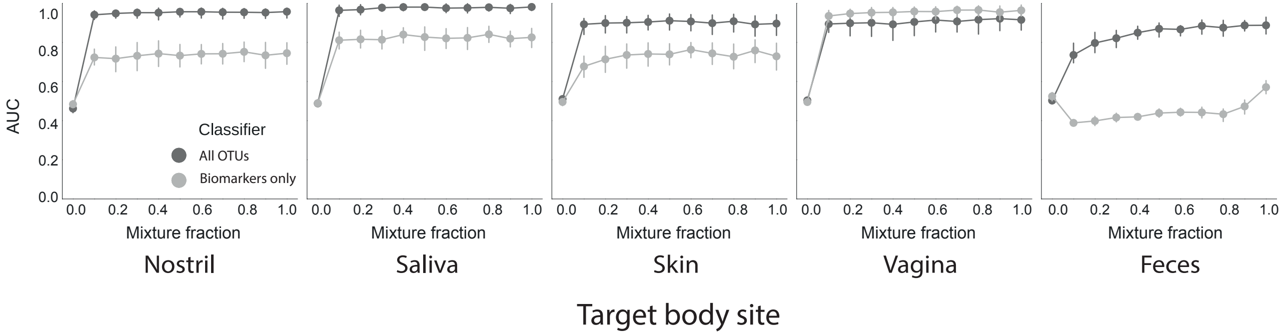

AUC
1.0
0.8
0.6
0.4
0.2
0.0
Classifier
All OTUs
Biomarkers only
0.0 0.2 0.4 0.6 0.8 1.0
Mixture fraction
Nostril
Saliva
Skin
Vagina
Feces
Target body site
