# Supplementary material for: Ecologically informed microbial biomarkers and accurate classification of mixed and unmixed samples in an extensive cross-study of human body sites
Source: Microbiome. 2018 Oct 24;6:192. doi: 10.1186/s40168-018-0565-6 (PMC6201589; doi:10.1186/s40168-018-0565-6)
Supplement: Supplementary file 13 — Table S1. Top 5 most important positively associated biomarker genera per body site, measured by maximum feature importance in RFC-global among biomarker OTUs of each genus. (DOCX 7 kb) [file 40168_2018_565_MOESM13_ESM.docx]

| **Body site** | **Genus** | **Number of positively associated**  **OTUs** | **Percentile of Random Forest feature importance** |
| --- | --- | --- | --- |
|  | *Corynebacterium* | 6 | 100.0 |
|  | *Cutibacterium* | 2 | 99.8 |
| Nostril | *Dolosigranulum* | 1 | 93.2 |
|  | *Acinetobacter* | 1 | 90.1 |
|  | *Micrococcus* | 1 | 85.0 |
|  | *Veillonella* | 2 | 99.3 |
|  | *Prevotella* | 8 | 99.2 |
| Saliva | *Neisseria* | 1 | 98.9 |
|  | *Actinomyces* | 1 | 98.1 |
|  | *Gemella* | 2 | 98.0 |
|  | *Corynebacterium* | 2 | 100.0 |
|  | *Cutibacterium* | 2 | 99.8 |
| Skin | *Streptococcus* | 3 | 99.1 |
|  | *Neisseria* | 1 | 98.9 |
|  | *Gemella* | 1 | 98.0 |
|  | *Lactobacillus* | 6 | 98.3 |
|  | *Prevotella* | 3 | 95.7 |
| Vagina | *Finegoldia* | 1 | 94.6 |
|  | *Gardnerella* | 1 | 94.5 |
|  | *Atopobium* | 2 | 93.1 |
|  | *Bacteroides* | 21 | 93.9 |
|  | *Enterococcus* | 2 | 88.8 |
| Feces | *Parabacteroides* | 3 | 88.3 |
|  | *Eubacterium* | 4 | 86.5 |
|  | *Clostridium* | 5 | 82.2 |
