# Supplementary material for: Ecologically informed microbial biomarkers and accurate classification of mixed and unmixed samples in an extensive cross-study of human body sites
Source: Microbiome. 2018 Oct 24;6:192. doi: 10.1186/s40168-018-0565-6 (PMC6201589; doi:10.1186/s40168-018-0565-6)
Supplement: Supplementary file 14 — Table S2. Novel and previously described positive associations between genera and body sites. (DOCX 23 kb) [file 40168_2018_565_MOESM14_ESM.docx]

| **Body site** | **Genus** | **Number of positively associated OTUs** | **Reference** |
| --- | --- | --- | --- |
| Nostril | Corynebacterium | 6 | [1] |
|  | Cutibacterium | 2 | [2] |
|  | Acinetobacter | 1 | [3] |
|  | Delftia | 1 | not described in nostril |
|  | Dolosigranulum | 1 | [4] |
|  | Micrococcus | 1 | [4] |
| Saliva | Prevotella | 8 | [5] |
|  | Selenomonas | 2 | [5] |
|  | Capnocytophaga | 4 | [5] |
|  | Actinomyces | 1 | [5] |
|  | Gemella | 2 | [5] |
|  | Leptotrichia | 2 | [5] |
|  | Veillonella | 2 | [5] |
|  | Fusobacterium | 1 | [5] |
|  | Haemophilus | 1 | [5] |
|  | Streptococcus | 1 | [5] |
|  | Atopobium | 1 | [6] |
|  | Rothia | 1 | [5] |
|  | Peptostreptococcus | 1 | [6] |
|  | Campylobacter | 1 | [5] |
|  | Lautropia | 1 | [7] |
|  | Catonella | 1 | [8] |
|  | Granulicatella | 1 | [5] |
|  | Porphyromonas | 1 | [5] |
|  | Neisseria | 1 | [5] |
|  | [Eubacterium] sulci | 1 | [8] |
|  | Mogibacterium | 1 | [6] |
|  | Lachnoanaerobaculum | 1 | [9] |
| Skin | Streptococcus | 3 | [11] |
|  | Corynebacterium | 2 | [10] |
|  | Paracoccus | 1 | [11] |
|  | Gemella | 1 | [12] |
|  | Sphingomonas | 1 | [11] |
|  | Ralstonia | 1 | not described in skin |
|  | Pseudomonas | 1 | [13] |
|  | Moraxella | 2 | [12] |
|  | Micrococcus | 1 | [13] |
|  | Acinetobacter | 1 | [14] |
|  | Actinomyces | 1 | [15] |
|  | Delftia | 1 | [13] |
|  | Acidovorax | 1 | [11] |
|  | Cutibacterium | 2 | [2] |
|  | Neisseria | 1 | [11] |
|  | Caulobacter | 1 | not described in skin |
|  | Fusobacterium | 1 | [12] |
| Vagina | Lactobacillus | 6 | [16] |
|  | Anaerococcus | 3 | [17] |
|  | Prevotella | 3 | [16] |
|  | Atopobium | 2 | [16] |
|  | Aerococcus | 1 | [16] |
|  | Finegoldia | 1 | [16] |
|  | Sneathia | 1 | [16] |
|  | Mycoplasma | 1 | [18] |
|  | Mycobacterium | 1 | [19] |
|  | Actinomyces | 1 | [19] |
|  | Gardnerella | 1 | [16] |
|  | Ureaplasma | 1 | [18] |
|  | Veillonella | 1 | [20] |
| Feces | Bacteroides | 22 | [21] |
|  | Ruminiclostridium | 1 | [22] |
|  | Clostridium | 5 | [21] |
|  | Oscillibacter | 1 | [23] |
|  | Eubacterium | 4 | [22] |
|  | Alistipes | 3 | [24] |
|  | Tyzzerella | 2 | [25] |
|  | Lachnoclostridium | 5 | [22] |
|  | Ruminococcus | 3 | [21] |
|  | Bifidobacterium | 4 | [21] |
|  | Faecalibacterium | 1 | [21] |
|  | Roseburia | 1 | [23] |
|  | Parabacteroides | 3 | [24] |
|  | Acidaminococcus | 2 | [22] |
|  | Collinsella | 2 | [25] |
|  | Anaerotruncus | 1 | [26] |
|  | Weissella | 1 | [27] |
|  | Anaerostipes | 1 | [28] |
|  | Bilophila | 1 | [29] |
|  | Blautia | 5 | [30] |
|  | Parasutterella | 1 | [24] |
|  | Odoribacter | 1 | [28] |
|  | Dorea | 2 | [23] |
|  | Lactococcus | 1 | [31] |
|  | Coprococcus | 1 | [22] |
|  | Akkermansia | 1 | [26] |
|  | Erysipelatoclostridium | 3 | [22] |
|  | Enterococcus | 2 | [21] |
|  | putative Halovenus | 1 | not described in gut |
|  | Holdemanella | 2 | [32] |
|  | Oxalobacter | 1 | [33] |
|  | Salmonella | 1 | [34] |
|  | Lactococcus | 1 | [31] |
|  | Lactobacillus | 1 | [21] |
|  | Microvirgula | 1 | [35] |
|  | Paraclostridium | 1 | [36] |

## References

[1] Frank DN, Feazel LM, Bessesen MT, et al. The human nasal microbiota and Staphylococcus aureus carriage. *PLoS One* 2010; 5: e10598.

[2] Fitz-Gibbon S, Tomida S, Chiu B-H, et al. Propionibacterium acnes strain populations in the human skin microbiome associated with acne. *J Invest Dermatol* 2013; 133: 2152–2160.

[3] Mahdavinia M, Keshavarzian A, Tobin MC, et al. A comprehensive review of the nasal microbiome in chronic rhinosinusitis (CRS). *Clin Exp Allergy* 2016; 46: 21–41.

[4] Kaspar U, Kriegeskorte A, Schubert T, et al. The culturome of the human nose habitats reveals individual bacterial fingerprint patterns. *Environ Microbiol* 2016; 18: 2130–2142.

[5] Li J, Quinque D, Horz H-P, et al. Comparative analysis of the human saliva microbiome from different climate zones: Alaska, Germany, and Africa. *BMC Microbiol* 2014; 14: 316.

[6] Gomes BPFA, Berber VB, Kokaras AS, et al. Microbiomes of Endodontic-Periodontal Lesions before and after Chemomechanical Preparation. *J Endod* 2015; 41: 1975–1984.

[7] Nasidze I, Li J, Quinque D, et al. Global diversity in the human salivary microbiome. *Genome Res* 2009; 19: 636–643.

[8] Lazarevic V, Gaïa N, Girard M, et al. Comparison of DNA extraction methods in analysis of salivary bacterial communities. *PLoS One* 2013; 8: e67699.

[9] Johansson I, Witkowska E, Kaveh B, et al. The Microbiome in Populations with a Low and High Prevalence of Caries. *J Dent Res* 2016; 95: 80–86.

[10] Costello EK, Lauber CL, Hamady M, et al. Bacterial community variation in human body habitats across space and time. *Science* 2009; 326: 1694–1697.

[11] Cosseau C, Romano-Bertrand S, Duplan H, et al. Proteobacteria from the human skin microbiota: Species-level diversity and hypotheses. *One Health* 2016; 2: 33–41.

[12] Oh J, Freeman AF, NISC Comparative Sequencing Program, et al. The altered landscape of the human skin microbiome in patients with primary immunodeficiencies. *Genome Res* 2013; 23: 2103–2114.

[13] Grice EA, Kong HH, Renaud G, et al. A diversity profile of the human skin microbiota. *Genome Res* 2008; 18: 1043–1050.

[14] Smeekens SP, Huttenhower C, Riza A, et al. Skin microbiome imbalance in patients with STAT1/STAT3 defects impairs innate host defense responses. *J Innate Immun* 2014; 6: 253–262.

[15] van Rensburg JJ, Lin H, Gao X, et al. The Human Skin Microbiome Associates with the Outcome of and Is Influenced by Bacterial Infection. *MBio* 2015; 6: e01315–15.

[16] Ravel J, Gajer P, Abdo Z, et al. Vaginal microbiome of reproductive-age women. *Proc Natl Acad Sci U S A* 2011; 108 Suppl 1: 4680–4687.

[17] Zhou X, Brown CJ, Abdo Z, et al. Differences in the composition of vaginal microbial communities found in healthy Caucasian and black women. *ISME J* 2007; 1: 121–133.

[18] Marrazzo JM, Koutsky LA, Eschenbach DA, et al. Characterization of vaginal flora and bacterial vaginosis in women who have sex with women. *J Infect Dis* 2002; 185: 1307–1313.

[19] Huppert JS, Bates JR, Weber AF, et al. Abnormal vaginal pH and Mycoplasma genitalium infection. *J Pediatr Adolesc Gynecol* 2013; 26: 36–39.

[20] Redondo-Lopez V, Cook RL, Sobel JD. Emerging role of lactobacilli in the control and maintenance of the vaginal bacterial microflora. *Rev Infect Dis* 1990; 12: 856–872.

[21] Palmer C, Bik EM, DiGiulio DB, et al. Development of the human infant intestinal microbiota. *PLoS Biol* 2007; 5: e177.

[22] Creevey CJ, Kelly WJ, Henderson G, et al. Determining the culturability of the rumen bacterial microbiome. *Microb Biotechnol* 2014; 7: 467–479.

[23] Raman M, Ahmed I, Gillevet PM, et al. Fecal microbiome and volatile organic compound metabolome in obese humans with nonalcoholic fatty liver disease. *Clin Gastroenterol Hepatol* 2013; 11: 868–75.e1–3.

[24] Shahinas D, Silverman M, Sittler T, et al. Toward an understanding of changes in diversity associated with fecal microbiome transplantation based on 16S rRNA gene deep sequencing. *MBio*; 3. Epub ahead of print 23 October 2012. DOI: 10.1128/mBio.00338-12.

[25] Joossens M, Huys G, Cnockaert M, et al. Dysbiosis of the faecal microbiota in patients with Crohn’s disease and their unaffected relatives. *Gut* 2011; 60: 631–637.

[26] Biagi E, Nylund L, Candela M, et al. Through ageing, and beyond: gut microbiota and inflammatory status in seniors and centenarians. *PLoS One* 2010; 5: e10667.

[27] Walter J, Hertel C, Tannock GW, et al. Detection of Lactobacillus, Pediococcus, Leuconostoc, and Weissella species in human feces by using group-specific PCR primers and denaturing gradient gel electrophoresis. *Appl Environ Microbiol* 2001; 67: 2578–2585.

[28] Morgan XC, Tickle TL, Sokol H, et al. Dysfunction of the intestinal microbiome in inflammatory bowel disease and treatment. *Genome Biol* 2012; 13: R79.

[29] Smith MI, Yatsunenko T, Manary MJ, et al. Gut microbiomes of Malawian twin pairs discordant for kwashiorkor. *Science* 2013; 339: 548–554.

[30] Rajilić–Stojanović M, Biagi E, Heilig HGHJ, et al. Global and Deep Molecular Analysis of Microbiota Signatures in Fecal Samples From Patients With Irritable Bowel Syndrome. *Gastroenterology* 2011; 141: 1792–1801.

[31] Barrett E, Kerr C, Murphy K, et al. The individual-specific and diverse nature of the preterm infant microbiota. *Arch Dis Child Fetal Neonatal Ed* 2013; 98: F334–40.

[32] Tanca A, Abbondio M, Palomba A, et al. Potential and active functions in the gut microbiota of a healthy human cohort. *Microbiome* 2017; 5: 79.

[33] Stewart CS, Duncan SH, Cave DR. Oxalobacter formigenes and its role in oxalate metabolism in the human gut. *FEMS Microbiol Lett* 2004; 230: 1–7.

[34] Ahmer BMM, Gunn JS. Interaction of Salmonella spp. with the Intestinal Microbiota. *Front Microbiol* 2011; 2: 101.

[35] Giamarellos-Bourboulis E, Tang J, Pyleris E, et al. Molecular assessment of differences in the duodenal microbiome in subjects with irritable bowel syndrome. *Scand J Gastroenterol* 2015; 50: 1076–1087.

[36] Tidjani Alou M, Million M, Traore SI, et al. Gut Bacteria Missing in Severe Acute Malnutrition, Can We Identify Potential Probiotics by Culturomics? *Front Microbiol* 2017; 8: 899.
