# Supplementary material for: Ecologically informed microbial biomarkers and accurate classification of mixed and unmixed samples in an extensive cross-study of human body sites
Source: Microbiome. 2018 Oct 24;6:192. doi: 10.1186/s40168-018-0565-6 (PMC6201589; doi:10.1186/s40168-018-0565-6)
Supplement: Supplementary file 17 — Text S1. Supplementary methods and analyses. (DOCX 21 kb) [file 40168_2018_565_MOESM17_ESM.docx]

**Supplementary Information**

**Optimal threshold estimation for the minimum number of OTUs per sample**

We computed the pairwise unweighted Jaccard similarity between all samples to determine the closest neighbors of each sample in terms of OTU composition. Next, we calculated the entropy of body site labels amongst the top 100 closest neighbors of each sample in order to identify cases in which samples from multiple body sites show high similarity and are thereby hard to distinguish. We found a strong negative correlation between the body site entropy of closest neighbors and the number of unique OTUs per sample, which indicates that samples with low numbers of unique OTUs (usually due to low sequencing depth) from different body sites look similar in terms of OTU content. We identified the optimal threshold of unique OTUs at which this correlation disappeared (approximately 20 unique OTUs per sample) and applied this threshold to exclude noisy samples from our analysis.

**Impact of unassignable reads on biomarker detection**

OTUs assigned to *Staphylococcus*, a genus commonly reported as abundant skin inhabitant, could only be identified at comparatively low relative abundance and prevalence among skin samples in GlobalBodysites (5e-5 to 7e-5 abundance in 47 to 197 samples, calculated across all *Staphylococcus* OTUs univariately associated to skin). Upon further investigation, we found this to be caused by many reads confidently hitting this genus in the taxonomy database, but not mapping to a single 96% OTU in the reference database, but rather multiple OTUs with comparable alignment scores, making exact an OTU assignment impossible.

To evaluate the impact of these missing reads on biomarker detection, we created an abundance profile with reads that directly map to the *Staphylococcus* genus (0.24 average abundance across 3'247 skin samples) and applied GLL on this profile. We found a direct association pattern between the *Staphylococcus* genus and skin, showing that despite the advantages of the 96% OTU definition (see discussion in the main text), it can lead to missing some biomarkers at more general taxonomic levels.

We also observed low relative abundance and prevalence for *Methanobrevibacter* in feces: OTUs assigned to this genus where found with lower abundance and prevalence than expected. Contrary to *Staphylococcus* however, the genus abundance profile analysis with GLL discarded *Methanobrevibacter* as direct biomarker, matching results from the OTU level analysis and showing that unassignable reads do not always confound biomarker detection.

As an additional note, we generally expect the usage of presence-absence normalization for biomarker detection as done in this study to reduce the effect of unassignable reads due to inherent robustness to noise in abundances.
